# Regulation of cell cycle in plant gametes: when is the right time to divide?

**DOI:** 10.1242/dev.204217

**Published:** 2025-01-20

**Authors:** Sara Simonini

**Affiliations:** Department of Plant and Microbial Biology, University of Zurich, Zollikerstrasse 107, CH8008, Zurich, Switzerland

**Keywords:** Cell cycle, Fertilization, Plant gametes

## Abstract

Cell division is a fundamental process shared across diverse life forms, from yeast to humans and plants. Multicellular organisms reproduce through the formation of specialized types of cells, the gametes, which at maturity enter a quiescent state that can last decades. At the point of fertilization, signalling lifts the quiescent state and triggers cell cycle reactivation. Studying how the cell cycle is regulated during plant gamete development and fertilization is challenging, and decades of research have provided valuable, yet sometimes contradictory, insights. This Review summarizes the current understanding of plant cell cycle regulation, gamete development, quiescence, and fertilization-triggered reactivation.

## Teaser

**Summary:** This Review summarizes the current understanding of plant gamete development, cell cycle regulation, quiescence, and fertilization-triggered reactivation.

## Introduction

**Fig. 1.**
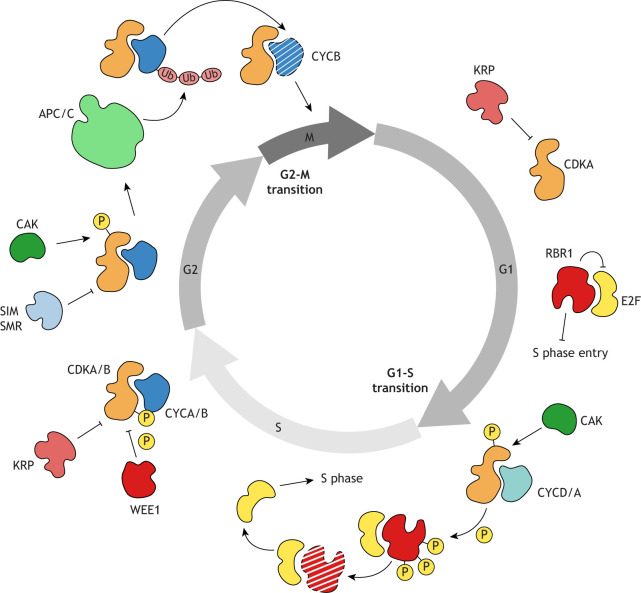
**The cell cycle in *Arabidopsis.*** Graphical representation of the *Arabidopsis* cell cycle and of its regulators involved in each phase. The phases of the cell cycle (G1-S-G2-M) are represented with arrows, reflecting the unidirectionality of the process. High levels of the cell cycle inhibitors KRPs and RBR1 cause the cell cycle to arrest in the G1 phase by inhibiting CDKA activity and sequestering E2F, thus preventing E2F from activating the genes required for DNA replication. At the G1-S transition, levels of CYC D and A types increase, allowing the formation of CDKA–CYC complexes. With the activating phosphorylation from CAK, CDKA kinase activity is enhanced. CDKA then phosphorylates RBR1, leading to the proteasome-mediated degradation of RBR1. This releases E2F from inhibition, enabling the expression of genes necessary for DNA replication. At the S-G2 boundary, the cellular concentration of CYC A and B types increases, promoting their dimerization with CDKA-B kinases. The activity of the CDKA-B kinases is inhibited by KRPs, and by phosphorylation by WEE1. In the G2 phase, CDKA-B activity is further suppressed by the SIM/SMR inhibitors. However, as the cell prepares for mitosis, phosphorylation of CDKA-B by CAK activates the CDKA-B–CYCB complex, which in turn stimulates the activity of the APC/C complex. The APC/C mediates the ubiquitination of A- and B-type CYCs, leading to their degradation. The destruction of CYCB allows the cell to commit to mitosis. APC/C, ANAPHASE PROMOTING COMPLEX; CDKA, CDKB, CYCLIN-DEPENDENT KINASE A/B type; E2F, E2F TRANSCRIPTION FACTOR; KRP, KIP-RELARED PROTEIN; RBR1, RETINOBLASTOMA RELATED 1; SIM/SMR, SIAMESE/SIAMESE-RELATED; CAK, CDK-ACTIVATING KINASE; WEE1, WEE1 KINASE HOMOLOGUE; P, phosphorylation; Ub, ubiquitination.

The cell is the basic structural and functional unit of life. Through cell division, new cells are produced, ensuring the continuity of life and supporting the development and function of all living beings. Cells typically divide following a unidirectional order of four cell cycle stages: the S (synthesis) phase, when cells replicate their nuclear DNA; the M (mitosis) phase, when sister chromatids separate and are allocated to the nascent daughter cells; and two gap phases, G1 and G2, that space the M and S phases (Fig. 1). Following these rules, two types of cell division can occur: mitosis and meiosis. During mitosis, diploid cells replicate their DNA content and, through one round of division, divide to produce two diploid daughter cells. Mitosis is typical of somatic cells and contributes to the growth, development and repair of tissues and organs. In contrast, meiosis is a process where cells replicate their genome, but then the DNA content is distributed among four cells through two consecutive chromosome separations. The resulting cells are therefore haploid and contain half of the genetic material of the mother cell. Meiosis is a type of cell division restricted to the cells of the germline, through which female and male gametes – eggs and sperm – are produced. Multicellular organisms that reproduce through sexual reproduction, such as animals and plants, have evolved sophisticated mechanisms to regulate cell cycle progression during the development of female and male gametes. Once formed, gametes must enter a quiescent state to prevent cell division in the absence of fertilization. In some species, quiescence can last for months or even years. At fertilization, quiescency must be lifted, to allow the newly formed embryo to divide and grow. This raises important fundamental questions: What are the molecular mechanisms that establish the quiescent state in the gametes? How do gametes recognize that fertilization has occurred and that the cell cycle must be resumed? How does this fundamental aspect of sexual reproduction vary across different species?

Studying gamete development and quiescence poses significant technical challenges. In animals like frogs, eggs are easily collected and have indeed provided foundational insights into gamete biology, including cell cycle regulation. In contrast, mammalian gametes often develop during fetal stages, making their collection in sufficient numbers for downstream analyses complicated. Techniques like *in vitro* fertilization have been instrumental in overcoming these barriers, enabling detailed investigations of gamete development. Plants produce hundreds of male gametes, which are easy to collect, but female gametes are much harder to access as they develop deep within multiple layers of maternal tissue, making their collection both difficult and time-consuming. This has resulted in a historical gap in understanding cell cycle regulation and quiescence in plant female gametes, though recent advances are beginning to uncover the signals that govern these processes, which brings us closer to engineering fertilization in plants to guarantee seed production in crops.

In this Review, I summarize the molecular mechanisms regulating the cell cycle in eukaryotic cells and highlight how these processes govern the formation of plant gametes. I also bring together both classic and recent, sometimes controversial, discoveries about plant cell cycle regulation around the moment of fertilization.

## The molecular basis of the cell cycle

The cell cycle has been extensively investigated, with research conducted across a broad range of organisms, including yeast, worms, insects, amphibians, mammals and plants. At the end of the 19th century, several scientists made groundbreaking contributions to our understanding of cell division. While observing stained cells of salamander embryos, the German biologist Walther Flemming described filamentous structures within the nucleus (Flemming, 1882). These structures were later termed ‘chromosomes’ by Wilhelm von Waldeyer-Hartz, from the Greek words for ‘colour’ (Khroma) and ‘body’ (Soma) (Waldeyer-Hartz, 1888). In the publication *Zellsubstanz, Kern und Zelltheilung*, Flemming drew the first detailed map of the sequential events happening during cell division, leading to chromosome separation (Flemming, 1882). Around the same time, Belgian embryologist Edouard Van Beneden was the first to observe and describe meiosis in animal egg cells (Van Beneden, 1883) and Polish botanist Eduard Strasburger described female gametophyte development and mitosis in plants (Strasburger, 1876).

Since these early discoveries, a collective research effort has yielded a comprehensive understanding of the cell cycle across the cellular, tissue and organism levels. We now know that the control of progression through the cell cycle phases relies on the irreversible modification of protein activity, mainly achieved through protein phosphorylation. One of the first major discoveries in the field dates back to the 1970s, when Yoshio Masui's pioneering work in frog oocytes led to the hypothesis of the existence of a factor, the Maturation Promoting Factor (MPF), that is sufficient and necessary to drive cell cycle progression through the consecutive phases (Masui and Markert, 1971). The MPF was revealed to be a protein complex, consisting of two protein kinases (with phosphorylation activity) and an activating partner. One of the kinases was initially described in budding yeast, where it was named Cdc2/Cdc28 (Hartwell et al., 1974; Nurse et al., 1976). Cdc2/Cdc28 is conserved across mammals and plants, which has led to the concept of a universal cell cycle engine (Hartwell et al., 1974; Nurse et al., 1976; Gutierrez, 2016, 2022).

Cdc2/Cdc28 is required to trigger the G1-S and G2-M transitions, and it is constitutively present in the cell (Hartwell et al., 1974; Nurse et al., 1976). To guarantee that Cdc2/Cdc28 is effective only under certain conditions and solely at the G1-S and the G2-M boundaries, its full activation relies on its dimerization with a partner protein. This partner protein was first identified by Tim Hunt's team, who termed it ‘cyclin’ because its expression fluctuates in a cyclical manner along the cell cycle phases (Draetta et al., 1989; Evans et al., 1983; Gautier et al., 1990; Labbé et al., 1989; Simanis and Nurse, 1986). The second kinase in the MPF complex, named Greatwall (in frog) or Mastl (microtubule-associated serine/threonine kinase-like, in mammals) (Castro and Lorca, 2018; García-Blanco et al., 2019; Gharbi-Ayachi et al., 2010; Mochida et al., 2010), was discovered later, and was identified as a key component necessary for mitosis progression (Gharbi-Ayachi et al., 2010; Mochida et al., 2010). Greatwall promotes correct timing and progression of mitosis by counteracting the activity of protein phosphatase 2A (PP2A), which acts to dephosphorylate mitotic substrates (Gharbi-Ayachi et al., 2010; Mochida et al., 2010). Plants lack homologs of the Greatwall kinase but possess MAST kinases, which feature a catalytic domain similar to that of Greatwall (Chudinova et al., 2017; Karpov et al., 2010). However, it remains to be determined whether these proteins also share functional homology with Greatwall (Chudinova et al., 2017; Karpov et al., 2010).

## Cell cycle regulation in *Arabidopsis*

In plants, the homologues of yeast Cdc2/Cdc28 are known as CYCLIN-DEPENDENT KINASES (CDKs). CDKs are Serine-Threonine kinases that can phosphorylate a range of targets (Nigg, 1995). In this Review, unless otherwise stated, the data discussed come from *Arabidopsis* studies. The model plant *Arabidopsis thaliana* possesses at least 29 CDK and 15 CDK-like (CDKL) genes, which can be categorized into eight classes: CDKs from A- to G-type, and CDKLs (Joubès et al., 2000; Vandepoele et al., 2002). CDKA is the equivalent of the canonical Cdc2 in fission yeast and is directly involved in cell cycle progression at the G1-to-S and G2-to-M phase transitions, whereas the activity of the B-type CDKs (there are four B-type CDKs in *Arabidopsis*; Joubès et al., 2000) seems to be restricted to the late G2-M phase (Nowack et al., 2012).

During cell cycle progression, CDKs are activated by binding to a cyclin (CYCs, Fig. 1). In *Arabidopsis*, at least 50 CYCs have been identified and grouped into ten classes: A-, B-, C-, D-, H-, L-, SDS-, Q-, T- and P-types (Wang et al., 2004). The heterodimerization of CYCs with CDKs is required for the activation of CDK kinase activity (Arellano and Moreno, 1997). Some CYCs possess a destruction box (Dbox), which is recognized by ubiquitin-protein ligases and ultimately serves as a signal for their proteosomal-mediated degradation (Glotzer et al., 1991). The progression through the consecutive phases of the cell cycle relies on the regulated synthesis and waves of destruction of CYCs, which modulate the activation status of CDKs (Arellano and Moreno, 1997). These waves play a crucial role in driving the G1-S and G2-M transitions (Bähler, 2005). Various classes of CDKs and CYCs form diverse complexes in *Arabidopsis*, providing multi-layered regulation of cell cycle progression in response to external stimuli, developmental stage, and tissue and cell identity (Van Leene et al., 2010).

To ensure that CDKs are activated only when CYC concentrations reach certain thresholds, proteins belonging to the Kip-related proteins/Interactor-Inhibitor of Cyclin-Dependent Kinase (KRP/ICK) family (seven in *Arabidopsis*; De Veylder et al., 2001; Lui et al., 2000; Wang et al., 1997), as well as members of the SIAMESE/SIAMESE RELATED (SIM/SMR) family (17 in *Arabidopsis*; Churchman et al., 2006; Peres et al., 2007), inhibit CDK activity by binding to and physically blocking the catalytic site of CDKs (Fig. 1). Degradation of KRPs through ubiquitination and subsequent proteosome-mediated degradation lifts their inhibitory effect (Hengst, 2004; Verkest et al., 2005). Indeed, KRP degradation is essential to ensure mitotic progression (Liu et al., 2008; Ren et al., 2008).

The activity of CDKs is also regulated by phosphorylation. In yeast, animals and plants, CDK-activating kinase (CAK) phosphorylates a conserved threonine residue in the CDKs, thus enhancing accessibility of the CDK catalytic site (Dissmeyer et al., 2007; Harashima et al., 2007; Umeda et al., 2005). The first plant CAK orthologue was identified in rice and later named *Oryza sativa* CYCLIN DEPENDENT KINASE D;1 (Os;CDKD;1; Hata, 1991). In *Arabidopsis*, there are three CDKD genes: *CDKD;1*, *CDKD;2* and *CDKD;3* (Shimotohno et al., 2003). CDKDs are in turn phosphorylated and activated by the kinase CYCLIN-DEPENDENT KINASE F;1 (CDKF;1; Shimotohno et al., 2004; Takatsuka et al., 2009).

In animals and yeast, the kinase WEE1 negatively regulates CDK activity through phosphorylation, thereby inhibiting ATP binding and substrate recognition (McGowan and Russell, 1993; Parker and Piwnica-Worms, 1992). Plants possess WEE1 homologues (Gonzalez et al., 2004; Sorrell et al., 2002; Sun et al., 1999), which share some similarities with their animal counterparts but also exhibit distinct functions. In *Arabidopsis*, WEE1 phosphorylates CDKA;1 (De Schutter et al., 2007), similar to the WEE1-CDK interaction seen in animals. However, unlike in animals, this phosphorylation does not appear to impact CDKA;1 activity (Dissmeyer et al., 2010, 2009). In *Arabidopsis*, WEE1 can also inhibit cell cycle progression by directly interacting with and phosphorylating the E3 ubiquitin ligase F-BOX-LIKE 17 (FBL17) (Pan et al., 2021), which normally acts as an inhibitor of KRPs (Gusti et al., 2009; Kim et al., 2008; Zhao et al., 2012). Upon phosphorylation by WEE1, FBL17 becomes polyubiquitinated and is degraded, leading to the accumulation of KRPs and ultimately halting CDK activity and cell cycle progression (Pan et al., 2023, 2021).

These cell cycle components – CDKs, CYCs and inhibitors – operate in feedback loops so that each factor promotes the expression and stability of its activators, while repressing the activity of its inhibitors (Venta et al., 2012). This behaviour makes the cell cycle a robust, wired mechanism that generates two stable, opposite steady states: inactive and active (Harashima et al., 2013; Stallaert et al., 2019). The transition from one state to the other occurs as a biological switch (Ferrell, 2002; Pomerening et al., 2003). This property ensures the unidirectional progression of the cell cycle, making the system resistant to small fluctuations in protein concentrations because it takes higher levels of CDK activity (and CYC concentrations) to initiate mitosis than those required to maintain it (Harashima et al., 2013). This characteristic is defined as hysteresis, which is when a system requires more of a signal to switch states than to stay in the current state (Dragoi et al., 2024; Pomerening et al., 2003; Sha et al., 2003).

### The G1-S transition in *Arabidopsis*

The G1-S transition in *Arabidopsis* is primarily controlled by A- and D-type CYCs (Oakenfull et al., 2002; Takahashi et al., 2010). At the G1-S transition, the main target of the CDKA–CYCD complex is the homologue of the animal Retinoblastoma protein (pRb), a tumour suppressor protein, the dysfunction of which is often associated with tumorigenesis and cancer progression (Friend et al., 1986; Fung et al., 1987; Lee et al., 1987). pRb homologues have been identified in plants, including RBR1 in *Arabidopsis* (Ach et al., 1997a; Grafi et al., 1996; Kong et al., 2000; Nakagami et al., 1999; Xie et al., 1996). RBR1 exhibits remarkable functional conservation with the animal pRb (Ach et al., 1997a; Grafi et al., 1996; Kong et al., 2000; Nakagami et al., 1999; Xie et al., 1996). The main function of both animal pRb and plant RBR1 is to inhibit the progression of cells into S-phase by directly binding to E2F transcription factors, thus preventing E2F from activating genes necessary for DNA synthesis and cell cycle progression (Fig. 1) (Hiebert et al., 1992; Zamanian and La Thangue, 1992). In addition, similar to pRb in animals, plant RBR1 recruits chromatin-remodelling complexes such as histone methyltransferases and deacetylases to locally repress transcription (Brehm and Kouzarides, 1999; Gu et al., 2011; Jullien et al., 2008; Ötvös et al., 2021; Rossi et al., 2003; Vandel et al., 2001). D-type CYCs directly interact with pRb/RBR1 via the conserved LxCxE motif, thus tethering CDK activity to pRb/RBR1 (Dowdy et al., 1993; Ewen et al., 1993; Kato et al., 1993; Soni et al., 1995; Xie et al., 1995). CDK-dependent phosphorylation inhibits pRb/RBR1 dimerization with E2F and leads to its proteosomal-mediated degradation, thus promoting progression into S-phase (Burke et al., 2010; Harbour et al., 1999; Mittnacht et al., 1994; Mittnacht and Weinberg, 1991; Rubin et al., 2005) (Fig. 1). Reducing RBR1 levels can suppress the phenotype of the *cdka;1* mutant, suggesting that RBR1 is also the main target of CDKA;1 in *Arabidopsis* (Nowack et al., 2012; Ramírez-Parra et al., 1999; Sekine et al., 1999). RBR1 and pRb are also both involved in cell cycle arrest and DNA repair in response to DNA damage thanks to their ability to both halt DNA replication by blocking the recruitment or maintenance of crucial replication factors on chromatin, and to interact with DNA repair complexes (Knudsen et al., 1998, 2000; Angus et al., 2004; Biedermann et al., 2017; Horvath et al., 2017).

The concentration of G1-S inhibitors is also important for ensuring that cell division occurs appropriately according to the size of the cell. In large cells, cell cycle inhibitors become diluted, and this condition triggers the G1-S transition (D'Ario and Sablowski, 2019). Conversely, in small cells, the concentration of inhibitors remains high, prolonging the G1 phase to allow for cell growth before division (D'Ario and Sablowski, 2019). In human cells, the level of pRb correlates with the ability of a cell to divide: small cells contain a high concentration of pRb and thus their progression into the G1-S transition is inhibited, while large cells dilute pRb so that cell cycle progression is promoted (Zatulovskiy et al., 2020). A similar mechanism occurs in yeast, where the dilution of the pRb functional homologue Whi5 is dependent on cell growth (Schmoller et al., 2015). In plants, members of the KRP family bind to mitotic chromosomes, ensuring that daughter cells inherit equal amounts of the inhibitor, regardless of their size (D'Ario et al., 2021; Sablowski and Gutierrez, 2022). In smaller daughter cells, the higher concentration of KRPs delays entry into the S phase, allowing the cell to grow to the appropriate size during the G1 phase (D'Ario et al., 2021).

### The G2-M transition in *Arabidopsis*

The G2-M transition in *Arabidopsis* is governed by B-type CYCs, which, in complex with CDKs, phosphorylate several substrates to regulate processes including nuclear envelope breakdown, chromosome condensation, spindle assembly, microtubule organization and chromosome segregation (Blethrow et al., 2008; Menges et al., 2005; Romeiro Motta et al., 2022; Wang et al., 2004). To complete mitosis and promote cytokinesis, CYCB must be degraded by the Anaphase Promoting Complex/Cyclosome (APC/C, Fig. 1), a multi-subunit ubiquitin-ligase complex that recognises the Dbox in B-type CYCs, targeting them for degradation (Townsley and Ruderman, 1998). APC/C activation specificity relies on the coactivators CDC20 and CCS52B (Pesin and Orr-Weaver, 2008), the mRNAs of which are retained in the nucleus until nuclear envelope breakdown, when they are released into the cytoplasm to be translated (Yang et al., 2017).

The expression of genes specific to the G2/M transitions is regulated by a multimeric, evolutionarily conserved complex known as the DREAM (DP, Rb-like, E2F and MuvB) complex (Guiley et al., 2015; Sadasivam and DeCaprio, 2013). In animals, the DREAM complex is composed of E2F and Myb-type transcription factors and Rb-related proteins (Guiley et al., 2015). This complex is conserved across various species, including nematodes, fruit flies, animals and plants (Harrison et al., 2007, 2006; Kobayashi et al., 2015; Korenjak et al., 2004; Lewis et al., 2004; Litovchick et al., 2007; Pilkinton et al., 2007; Schmit et al., 2007). In plants, there are at least two distinct flavours of the DREAM complex, depending on the transcriptional activity of the MYB-type transcription factors (Kobayashi et al., 2015; Latorre et al., 2015; Magyar et al., 2016). DREAM complexes containing activator MYB3Rs are involved in promoting expression of mitotic genes, particularly those genes that are essential for cytokinesis (Haga et al., 2007). In contrast, repressor MYB3Rs function outside the mitotic phase, playing a key role in silencing mitotic genes and imposing quiescence in mature organs (Kobayashi et al., 2015).

## Cell cycle regulation during plant reproduction

Gametes – sperm in males and eggs in females – are specialized cells that are haploid at maturity. Once fully developed, gametes enter a state of quiescence, which is lifted by signals occurring at fertilization, enabling the zygote to initiate mitotic cycles (Tao and Nodine, 2019; Khanday et al., 2023). Precisely orchestrated cell cycle events govern gamete development, the acquisition of quiescence, and exit from quiescence at fertilization. Failures in any of these processes can lead to developmental defects, potentially causing abortion of the progeny (Chaudhury et al., 1997; Guitton and Berger, 2005).

Gametogenesis evolved independently in animals and plants (Barrett, 2002). In animals, functional gametes are the direct product of meiosis of primordial germ cells, which are specified during early embryogenesis (Saffman and Lasko, 1999). Spermatogenesis begins and proceeds continuously postnatally, whereas oogenesis initiates during foetal development and mature oocytes await fertilization at arrested meiosis II (Pei et al., 2023). In contrast, the life cycle of land plants alternates between two generations: the sporophyte and the gametophyte (Pandey et al., 2022). The sporophyte is the diploid phase, through which haploid spores are produced by meiosis (Hisanaga et al., 2019). During the gametophytic phase, these haploid spores undergo mitosis to produce the female and male gametes (Hisanaga et al., 2019). The structure that harbours the gametes is called gametophyte; pollen is the gametophyte for the male gametes (Twell, 2011), and the embryo sac is the gametophyte for the female gametes (Drews and Koltunow, 2011). The dominance of either the gametophytic or sporophytic phase varies by species: in mosses, the gametophyte is dominant, whereas in vascular plants, such as gymnosperms and angiosperms such as *Arabidopsis*, the sporophyte is predominant (Hisanaga et al., 2019). The transitions between these phases happen at two developmental points: fertilization, where two haploid gametes fuse to form a diploid sporophyte, and meiosis, where the diploid sporophyte generates haploid gametophytes. Thus, in flowering plants, gametes are not the direct products of meiosis. Instead, haploid spores formed after meiosis undergo additional rounds of mitosis to produce the gametes (Berger and Twell, 2011; Dresselhaus et al., 2016). The male gametes (sperm cells) arise from two rounds of mitosis (Twell, 2011), while the female gametes (comprising the egg cell and central cell) result from three rounds of mitosis (Drews and Koltunow, 2011).

In angiosperms, reproductive structure development and gametogenesis typically occur after the plant has undergone a vegetative phase during which only vegetative tissues, such as leaves, are produced, and no reproductive structures are formed (Araki, 2001). Upon receiving external signals, such as changes in light and temperature, as well as internal cues such as hormone levels, the vegetative meristem transitions into an inflorescence meristem (Chahtane et al., 2023). In plants like *Arabidopsis*, this identity change marks the plant's irreversible commitment to the reproductive phase. Flowers arise from the flanks of the apical meristem, starting as a small, undifferentiated cluster of cells (Smyth et al., 1990). During development, the floral organs are specified and differentiate into flowers. The mature *Arabidopsis* flower consists of four concentric whorls: from the outside in, these are four sepals, four petals, six stamens, and a central pistil made of two fused carpels (Alvarez-Buylla et al., 2010). At the tip of each stamen, the anthers are the site of male gametophyte (pollen) development. Inside the pistil, ovules house the female gametophyte (the embryo sac). Within these male and female gametophytes, the germline cells develop as sperm in pollen and as the egg cell and central cell in the embryo sac (Berger and Twell, 2011).

### Male reproductive development

**Fig. 2.**
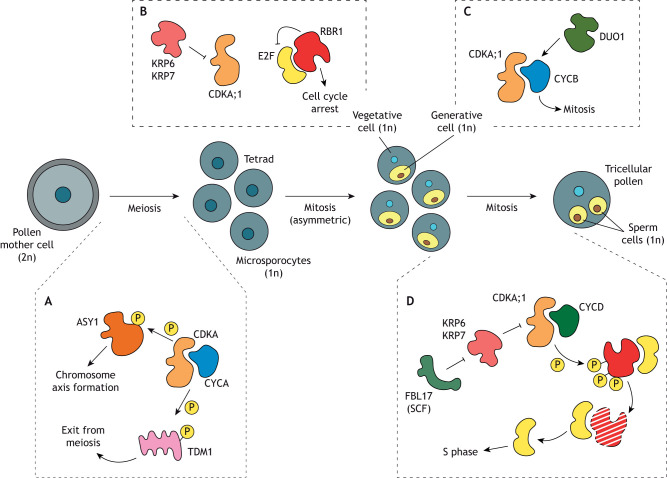
**Male reproductive development in *Arabidopsis.*** (A-D) Graphical summary of the main events during *Arabidopsis* male reproductive development, and of the cell cycle regulators involved in the meiosis and mitosis events. Pollen development begins in the anthers, where diploid pollen mother cells undergo meiosis to produce four haploid microspores. During meiosis (A), CDKA–CYC complexes phosphorylate a series of target proteins, such as ASY1 and TDM1, which control chromosome axis formation and exit from meiosis. During male gametogenesis, the microspores undergo an asymmetric mitotic division, producing a large vegetative cell and a smaller generative cell. Cell division in the vegetative cell is inhibited by RBR1 and KRPs (B). The generative cell undergoes another round of mitosis, resulting in two sperm cells. This process is mediated by DUO1, which activates the CDKA;1–CYCB complex (C). Both mitotic divisions are regulated by the CDKA;1–CYCD complex. FBL17 facilitates the degradation of KRPs, thereby enabling CDKA;1 activity during the second mitotic division. CDKA;1 activity, in turn, promotes the phosphorylation and degradation of RBR1, thus releasing E2F from inhibition and enabling the expression of genes necessary for DNA replication (D). ASY1, ASYNAPTIC 1; CDKA, CYCLIN-DEPENDENT KINASE A; CYCA/D, CYCLIN A/D-type; E2F, E2F TRANSCRIPTION FACTOR; KRP, KIP-RELATED PROTEIN; RBR1, RETINOBLASTOMA RELATED 1; DUO1, DUO POLLEN 1; FBL17, F-BOX LIKE 17; SCF, SCF ubiquitin ligase complex; TDM1, THREE DIVISION MUTANT 1; P, phosphorylation.

The mature male gametophyte (the pollen grain) contains the male gametes (the sperm cells) (Borg et al., 2009; McCormick, 2004). Pollen development begins when a sporogeneous cell undergoes meiosis to produce four haploid microspores (Fig. 2) (Borg et al., 2009; McCormick, 2004; Twell, 2011). The microspore then undergoes an asymmetric mitotic division to generate a large vegetative cell and a small, unique male generative cell (Fig. 2) (Borg et al., 2009; McCormick, 2004). This stage is defined as ‘bicellular pollen’. The generative cells then divide once more by mitosis to produce the two sperm cells, creating the final, mature male gametophyte (Borg et al., 2009; McCormick, 2004). A pollen with one vegetative cell and two sperm cells is defined as tricellular pollen (Fig. 2) (Borg et al., 2009; McCormick, 2004). Pollen grains from most angiosperms are held at the bicellular stage when mature, and the last mitosis event that produces the two sperm cells occurs after pollen germination within the pollen tube (Brewbaker, 1967). In other angiosperms such as *Arabidopsis* and rice, the mature pollen is tricellular because the last mitosis of the generative cell occurs before pollen germination (Berger and Twell, 2011; Brewbaker, 1967).

Below, I discuss the important roles that cycle components play in each step of pollen formation, from meiosis to asymmetric cell division, and mitosis.

#### Male sporogenesis

During meiosis, several types of CYCs are expressed in the microspore cells (Bulankova et al., 2013). A-type CYCs are involved in the correct segregation of meiotic chromosomes, and the timely entry and exit from meiosis (Bulankova et al., 2013; Cairo et al., 2022; Cifuentes et al., 2016; d'Erfurth et al., 2010) (Fig. 2A). For example, CYCA1;2 in complex with CDKA;1 phosphorylates THREE-DIVISION MUTANT 1 (TDM1; also known as MALE-STERILE 5), a tetratricopeptide repeat protein that controls meiotic exit by suppressing translation (Cairo et al., 2022; Cifuentes et al., 2016; d'Erfurth et al., 2010). Moreover, CDKA;1 directly phosphorylates the chromosome axis protein ASYNAPTIC 1 (ASY1), which is involved in the formation of the chromosome axis during meiosis (Yang et al., 2020).

The activity of CYCB3;1, a B-type CYC, is required during meiosis I, where it localizes to the meiotic spindles to repress the premature onset of cell wall formation (Bulankova et al., 2013; Sofroni et al., 2020). This function is shared by the cyclin-related factor SOLO DANCERS (SDS), a highly divergent cyclin, the expression of which is restricted to male and female meiotic cells, where it is required for meiotic recombination and chromosome pairing (Azumi et al., 2002). SDS also forms a complex with CDKA;1 to phosphorylate SWITCH1 (SWI1), leading to SWI1 degradation (Yang et al., 2019). SWI1 inhibits WINGS APART-LIKE (WAPL), so the degradation of SWI1 allows WAPL to perform its function of removing cohesin from chromosomes (Yang et al., 2019).

#### Male gametogenesis

After meiosis, each haploid microspore undergoes mitosis to generate the vegetative cell and the germ cell (Twell, 2011). In *Arabidopsis*, the acquisition of vegetative cell identity relies on the activity of RBR1, and *rbr1* vegetative cells retain microspore features (such as the ability to divide symmetrically) (Chen et al., 2009). The vegetative cell must arrest its cell cycle to prevent further divisions, and this process is governed by the cell cycle inhibitors RBR1 and KRPs (likely KRP1,3,4,6,7) (Chen et al., 2009; Kim et al., 2008; Zhao et al., 2012) (Fig. 2B). Meanwhile, germ cell identity is regulated by the germline-specific MYB transcription factor DUO1 POLLEN1 (DUO1), which activates CYCB1;1 to initiate mitosis (Brownfield et al., 2009) (Fig. 2C). Mutations in DUO1 lead to germ cells that are unable to divide, resulting in a significant proportion of blocked divisions (Brownfield et al., 2009).

The germ cell undergoes two mitotic divisions, which in *Arabidopsis* pollen are primarily regulated by CDKA;1 and factors modulating its activity (Aw et al., 2010; Iwakawa et al., 2006; Nowack et al., 2006; Zhao et al., 2012) (Fig. 2D). Heterozygous mutants of CDKA;1 develop pollen grains arrested at the bicellular state, where the vegetative cell develops properly but the generative cell fails to undergo mitosis to produce the two sperm cells (Iwakawa et al., 2006; Nowack et al., 2006). Interestingly, in some *cdka;1* mutant pollen grains, mitosis occurs after pollen germination and pollen tube elongation (Aw et al., 2010). Alongside CDKA;1, CDKB1;1 and CDKB1;2 also have a role in pollen development (Nowack et al., 2012). While pollen development in single and double *cdkb1;1 cdkb1;2* mutants is phenotypically similar to wild-type *Arabidopsis*, the triple mutant lacking CDKA;1, CDKB1;1 and CDKB1;2 activity (*CDKA;1/cdka;1 cdkb1;1/cdkb1;1 cdkb1;2/cdkb1;2*) produces a small proportion of pollen grains that fail to undergo any mitotic division (Nowack et al., 2012). The second mitotic division is also controlled by FBL17, which targets KRP6 and KRP7 for proteasome-mediated degradation (Gusti et al., 2009; Kim et al., 2008; Noir et al., 2015; Zhao et al., 2012). Mutations in *fbl17* lead to pollen arrest at the bicellular stage because the sustained levels of KRP6 and KRP7 inhibit CDKA;1 activity and thus cell cycle progression (Gusti et al., 2009; Kim et al., 2008; Zhao et al., 2012).

### Female reproductive development

In flowering plants, the mature female gametophyte contains two female gametes known as the egg cell and the central cell (Yadegari and Drews, 2004). During the process of double fertilization, two sperm cells fertilize the egg and the central cell to generate the zygote and the endosperm, a triploid tissue that provides nutrients to the embryo during its growth (Berger, 1999).

**Fig. 3.**
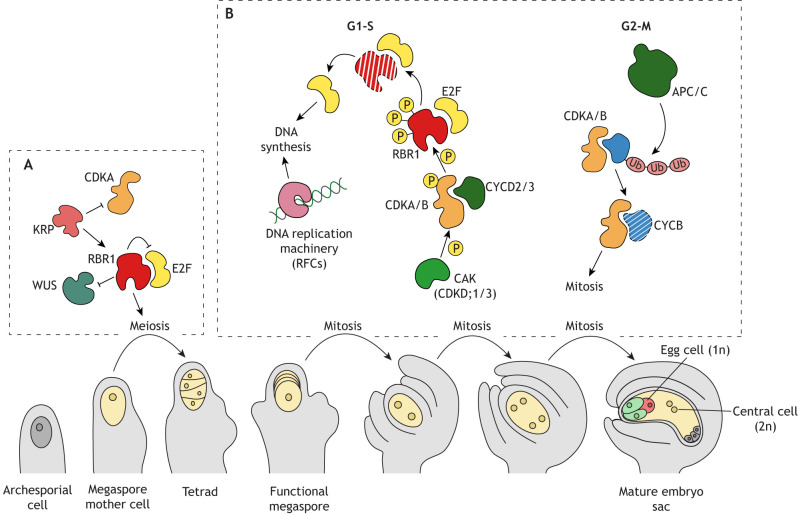
**Female reproductive development in *Arabidopsis.*** (A,B) Graphical summary of key events during *Arabidopsis* female reproductive development, and of the cell cycle regulators involved in the meiosis and mitosis events. The megaspore mother cell undergoes meiosis (A), forming a tetrad of haploid spores. KRPs inhibit CDKA;1, allowing RBR1 to accumulate and halt cell cycle progression by directly repressing *WUS* expression; this absence of WUS in the megaspore mother cell is crucial for limiting mitotic divisions and initiating meiosis. After meiosis, three spores degenerate, while the remaining spore undergoes three rounds of mitosis without cytokinesis (B). The mitotic divisions are orchestrated by CDKA;1–CYCD complexes during G1-S transitions and CDKA-B–CYCB complexes during G2-M transitions. Cellularization then takes place to produce one egg cell (magenta), two synergids (green), three antipodal cells (grey), and a central cell (yellow). The central cell is formed by the fusion of two haploid polar nuclei. APC/C, ANAPHASE PROMOTING COMPLEX; CDKA, CYCLIN-DEPENDENT KINASE A; CYCD, CYCLIN D-type; E2F, E2F TRANSCRIPTION FACTOR; CAK, CDK-ACXTIVATING KINASE; KRP, KIP-RELARED PROTEIN; RBR1, RETINOBLASTOMA RELATED 1; RFCs, REPLICATION FACTORS; WUS, WUSCHEL; P, phosphorylation; Ub, ubiquitination.

In *Arabidopsis*, female gametophyte development begins when an archesporial cell enlarges and differentiates into a megaspore mother cell (Grossniklaus and Schneitz, 1998; Schneitz et al., 1995; Yadegari and Drews, 2004). This cell, identifiable as a single, large and elongated subepidermal cell, undergoes meiosis to yield four megaspores, of which three undergo programmed cell death (Fig. 3) (Schneitz et al., 1995; Yadegari and Drews, 2004). The surviving megaspore develops into the functional megaspore, which undergoes three rounds of mitosis without cytokinesis to generate eight haploid nuclei (Fig. 3) (Grossniklaus and Schneitz, 1998; Yadegari and Drews, 2004). Cellularization ensues, culminating in the maturation of the female gametophyte: one egg cell, two synergid cells, three antipodal cells, and a central cell formed by the fusion of two haploid polar nuclei (Fig. 3) (Grossniklaus and Schneitz, 1998; Yadegari and Drews, 2004).

#### Female sporogenesis

Multiple subepidermal cells in the early ovule primordium possess the potential to become a megaspore mother cell; however, as the ovule develops, the number of cells capable of adopting such a fate is progressively reduced until only one cell attains the megaspore mother cell status (Grossniklaus and Schneitz, 1998). Positional cues, influenced by genetic and epigenetic networks and gradients of phytohormones, appear to orchestrate the specification of both the archesporial cell and the megaspore mother cell (Ceccato et al., 2013; She et al., 2013; Hernandez-Lagana et al., 2021; Huang et al., 2022). KRP4, KRP6 and KRP7 redundantly act within the megaspore mother cell to inhibit CDKA;1 activity so that RBR1 accumulates and represses cell cycle progression through direct suppression of *WUSCHEL *(*WUS*)** expression (Zhao et al., 2017; Fig. 3A). This absence of the transcription factor WUS in the designated megaspore mother cell is essential for limiting the number of mitotic divisions and triggering meiosis (Zhao et al., 2017). Indeed, female gametophytes lacking RBR1 or KRP function (such as in the *rbr1* mutant or the *krp1-7* septuple mutant) produce multiple megaspore mother cells, indicating that these megaspore mother cells are progressing into mitosis rather switching to the meiotic cycle (Cao et al., 2018; Zhao et al., 2017).

#### Female gametogenesis

Following megaspore mother cell specification, all the steps required to produce a functional female gametophyte are orchestrated by RBR1, CDKs and CYCs, and by the individual expression levels and protein turnover (by proteasome-mediated degradation) of these factors (Fig. 3B). In the triple mutant *CDKA;1/cdka;1 cdkb1;1/cdkb1;1 cdkb1;2/cdkb1;2*, megaspore mother cells can successfully undergo meiosis, but mitosis is severely compromised and ∼50% of embryo sacs arrest at various stages, meaning that they lack a cellularized egg apparatus and correct cellular identity (Nowack et al., 2012).

Lack of CDK activation by CYCs or CAKs also impairs female gametophyte development. Indeed, arrested development, tissue degeneration, and embryo sacs containing one, two, or four nuclei that fail to cellularize have been observed in multiple mutants for the D-type CYCs *cycd2;1 cycd3;3* (Zhang et al., 2022), in the absence of three or four B-type CYCs (*cycb1;1/cycb1;1 CYCB1;2/cycb1;2 cycb1;4/cycb1;4,* and *cycb1;1/cycb1;1 CYCB1;2/cycb1;2 CYCB1;3/cycb1;3 cycb1;4/cycb1;4*; Romeiro Motta et al., 2022), and in the double mutant of the two CAKs CDKD;1 and CDKD;3 (*cdkd;1-1 cdkd;3-1*, Takatsuka et al., 2015).

Progression of the megaspore mother cells through the mitotic cycle requires DNA synthesis, so absence of subunits of the DNA replication machinery such as REPLICATION FACTORS (RFCs) also impairs the division of the megaspore mother cell (Liu et al., 2013; Wang et al., 2012a,b).

Finally, mutations in some of the APC/C subunits also trigger female gametogenesis defects, because B-type CYCs are not efficiently degraded and thus mitosis cannot proceed normally (Pines, 2011). Phenotypes such as embryo sacs arrested at the one-cell stage, mis-positioned nuclei, abnormal nuclear number and degradation of the nuclei or embryo sac, have been observed in mutants for APC1 (Wang et al., 2013), APC2 (Capron, 2003), CDC27A and CDC27B (HOBBIT) (Blilou et al., 2002; Pérez-Pérez et al., 2008), APC4 (Wang et al., 2012a,b), NOMEGA (APC6) (Kwee and Sundaresan, 2003), APC13 (Saze and Kakutani, 2007) and APC10 (Eloy et al., 2011).

## Cell cycle stage in the mature gametes: awaiting fertilization

Regulation of cell cycle progression is also important to ensure that mature gametes are arrested at a cell cycle stage that will allow synchrony between male and female gametes when they meet at karyogamy (Friedman, 1999). Thus, attaining and maintaining quiescence is extremely important to prevent unwanted division in the gametes, a condition that can significantly impact an organism's fitness, waste energy and resources, and ultimately can lead to the abortion of progeny (Adhikari et al., 2010, 2009; Reddy et al., 2010).

In most animal species, mature sperm arrest in the G1 phase, whereas mature oocytes are arrested in metaphase of meiosis II (Griswold, 2016; Pei et al., 2023). In plants, the cell cycle phase at which gamete cells arrest varies by species, meaning that karyogamy in seed plants can take place in three different ways: G1 karyogamy, where the zygote DNA is replicated post-fusion; S-phase karyogamy, where the two nuclei go through S-phase before fusing; and G2 karyogamy, where nuclei fuse and then enter M-phase (Carmichael and Friedman, 1995).

**Fig. 4.**
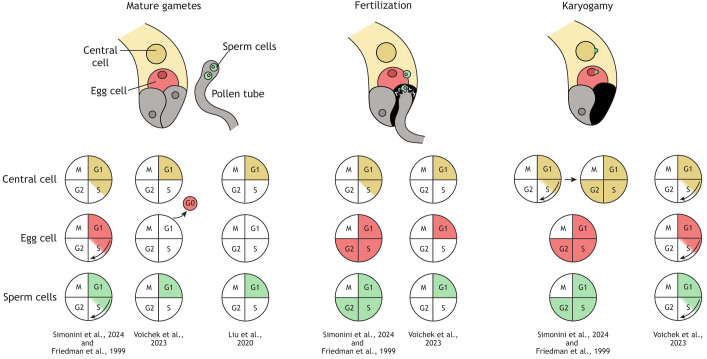
**Alternative models of the cell cycle stage of *Arabidopsis* gametes at maturity, fertilization and karyogamy.** Schematics summarizing alternative models (proposed by Friedman, 1999; Liu et al., 2020; Voichek et al., 2023 and Simonini et al., 2024) of the cell cycle stage of *Arabidopsis* female and male gametes at maturity, at the moment of fertilization and at karyogamy. Conflicting evidence has led to different models of the state of mature gametes (left panel): central cells (yellow) have been proposed to be in G1 (according to Liu et al. and Voichek et al.) or arrested S (according to Friedman et al. and Simonini et al.); egg cells (red) have been proposed to be in G0 (Voichek et al.), G1 (Liu et al.) or G2 (Friedman et al. and Simonini et al.); sperm cells (green) have been proposed to be in G1 (Liu et al. and Voichek et al.) or G2 (Friedman et al. and Simonini et al.). The progression of events during fertilization and karyogamy (middle and right panels) is closely tied to the cell cycle stages of the fusing gametes. If both gametes are in G1 or G0, DNA synthesis is initiated immediately after fusion, as suggested by studies such as Liu et al. and Voichek et al. For G2-phase gamete fusion, the fertilized gamete transitions directly into mitosis, as proposed in findings by Simonini et al. and Friedman et al. In scenarios where one gamete is in S-phase and the other is in G2, the S-phase gamete must first complete DNA replication to reach G2 before mitosis can proceed, a mechanism described in the context of the central cell by Simonini et al.

Classical techniques to determine the cell cycle stage of the gametes involve direct DNA staining methods such as DAPI, Propidium Iodide or Feulgen staining (Box 1). More recent strategies, such as feeding plants with fluorescently-labelled nucleotide analogues or analysing the dynamics of cell cycle component expression (e.g. CDT1a, PCNA, LIG1 and ORCs for S-phase, CYC B-type for mitosis), have further enhanced our understanding of DNA synthesis and cell cycle stages (Desvoyes et al., 2020; Echevarría et al., 2021). Numerous studies have sought to elucidate the cell cycle stage of mature plant gametes, with a historical emphasis on sperm cells due to their relative ease of isolation and analysis compared to the more challenging egg and central cells. Different research groups have reported conflicting results regarding the cell cycle stages of plant gametes, particularly in *Arabidopsis* (Table 1). For example, Friedman and others have reported that *Arabidopsis* sperm nuclei begin DNA synthesis soon after the second mitotic division of the generative cell (Durbarry et al., 2005; Friedman, 1999; Kim et al., 2008; Rotman et al., 2005), and that DNA replication continues as the sperms travel in the pollen tube, reaching the G2 stage with a 2C DNA content just before karyogamy (Table 1; Fig. 4). This conclusion is supported by gene expression studies showing that genes required for active DNA synthesis are expressed during pollen germination and pollen tube growth (Borges et al., 2008; Pina et al., 2005). Conversely, recent studies by Liu et al. and Voichek et al. found no evidence of DNA replication in mature sperm cells through EdU incorporation and DNA sequencing, suggesting that they do not enter S-phase at maturation (Liu et al., 2020; Voichek et al., 2023) (Table 1; Fig. 4). These conflicting results might be explained by the different techniques used. DAPI staining, employed in Friedman's study, may not reliably measure DNA content in sperm nuclei because of the extreme condensation of chromatin in these cells, potentially leading to inaccuracies in determining the cell cycle phase (Flores-Tornero and Becker, 2023; Liu et al., 2020). On the other hand, both the EdU staining and DNA sequencing techniques adopted in Liu et al. (2020) and Voichek et al. (2023) were conducted on sperm cells collected from pollen tubes grown *in vitro*, while DAPI staining by Friedman was done on whole flowers at various developmental stages. Pollen tubes grown by *in vitro* methods often fail to fertilize ovules, suggesting that the growth of the pollen tube through the female tissue is necessary for successful fertilization (Desnoyer and Grossniklaus, 2023). Therefore, semi*-in vitro* techniques might miss the ideal conditions for proper pollen activation and possibly also cell cycle progression.

**Box 1. Techniques used to assess the cell cycle stage of plant cells, including gametes**

**DAPI (4′,6-diamidino-2-phenylindole) staining:** DAPI is a very bright blue fluorescent DNA stain that associates with the minor groove of double-stranded DNA, with a preference for AT-rich regions. Its relative brightness matches to the abundance of nuclear DNA, and thus is used to quantify nuclear ploidy, from which the cell cycle stage is inferred. DAPI binds specifically to DNA and not RNA, so generates very little background signal. An example of applying this technique to *Arabidopsis* pollen is Borg et al. (2019).

**PI (Propidium Iodide) staining:** PI is a red-coloured DNA stain, which binds to double-stranded DNA by intercalating between base pairs. Its brightness is relative to the DNA content of a nucleus. PI binds nucleic acid in general, including RNA, so the staining procedure includes extensive RNase treatment to degrade RNA. An example of applying this technique to *Arabidopsis* ovules is She et al. (2013).

**Feulgen staining:** Feulgen staining specifically marks DNA. It liberates aldehydes from the deoxy sugar, allowing them to react with fuchsin-sulphurous acid and yield a magenta colour. However, chemicals present in plant tissues such as oils, resins, or tannins interfere with Feulgen staining detection, potentially leading to an overestimation of DNA quantity. An example of applying this technique to maize ovules is Barrell and Grossniklaus (2005).

**EdU (5-ethynyl-2′-deoxyuridine):** EdU is a nucleoside analogue of thymidine and is incorporated into newly synthesized DNA during S-phase. Its detection uses click-chemistry, where the EdU alkyne group undergoes a copper catalyzed click reaction with the azide group on an Alexa Fluor probe. EdU incorporation is used to assess the G1-S transition and S-phase. An example of applying this technique to *Arabidopsis* ovules is Simonini et al. (2024).

**DNA sequencing:** The quantity of DNA doubles from the G1 to the G2 phase, resulting in replicated genomic regions generating double the number of sequencing reads compared to non-replicated regions. If some genomic regions are doubled, it indicates that the cell is in the S phase. However, for this technique to be informative, it requires a comparison between non-replicated and replicated regions. DNA sequencing can provide evidence of DNA synthesis but not of ploidy, meaning it cannot distinguish whether a cell is in the G1 or G2 phase. An example of applying this technique to *Arabidopsis* pollen is Voichek et al. (2023).

**Marker lines:** Cell division can be monitored *in vivo* by visualizing the dynamics of constitutive nuclear markers, such as histone variants (H2B or H3) fused to fluorescent proteins. Specific cell cycle phases can also be detected by observing the accumulation, dynamics (e.g. diffuse signal versus speckles), or degradation of proteins that are specific to those phases. For example, expression of CYCB1;1 indicates mitosis, whereas PCNA condensation in speckles indicates the presence of replication foci during S phase, and CDT1a degradation happening at the G1-S boundary marks the beginning of G1-S transition. Some examples are found at Desvoyes et al. (2020), Echevarría et al. (2021), Voichek et al. (2023) and Simonini et al. (2024).

**Table 1.** Cell cycle stage of mature gametes before karyogamy

Plant species	Sperm cell cycle stage	Egg cell cycle stage	Central cell cycle stage	Reported in
Brown algae	G1	G1	–	McCully, 1968
Green algae	G1	G1	–	Matsuda et al., 1990
Red algae	G1	G1	–	Goff and Coleman, 1984
*Gnetum gnemon*	G2	G2	–	Carmichael and Friedman, 1995
*Chlorophytum elatum*	S	–	–	Ermakov et al., 1980
*Ligularia dentata*	S	–	–	Ermakov et al., 1980
*Crepis capillaris*	G2	–	–	Ermakov et al., 1980
*Elytrigia elongata*	G1	–	–	Ermakov et al., 1980
*Nuphar*	G1	–	–	Williams and Friedman, 2002
*Illicium*	G1	–	–	Williams and Friedman 2004
*Kadsura*	G1	–	–	Friedman et al., 2003
*Tradescantia paludosa*	G1	G1	–	Woodard, 1956
*Lycium barbarum*	G2	G2	–	Deng et al., 2012
*Helleborus bocconei*	G2	G2	G2	Bartoli et al., 2017
*Ginko biloba*	–	G2?	G2?	Avanzi and Cionini, 1971
*Vigna unguiculata*	–	G1	G2	Gursanscky et al., 2020 preprint
*Ornithogalum caudatum*	G2	G2	G1	Morozova, 2002
*Galanthus nivalis*	–	G1	–	Bannikova et al., 1985
*Allium odorum*	G2	G2	–	Morozova and Ermakov, 1993
*Arabidopsis thaliana*	G2	–	–	Friedman, 1999
	G1	G0	G1	Voichek et al., 2023
	G1	G1	G1	Liu et al., 2020
	G2	G2	Arrested S	Simonini et al., 2024
	–	G1	G2	Berger et al., 2008
*Zea mays*	G1	–	–	Bino et al., 1990
	G1	G1	–	Mogensen et al., 1995
	G1	G0	–	Chen et al., 2017
	G1	–	–	Swift, 1950
*Hordeum vulgare*	G1	G1	–	Mogensen and Holm, 1995
	–	G1	G1	Bennett and Smith, 1976
	G2	G2	–	D'Amato et al., 1965
*Torenia fournieri*	G1	–	–	Liu et al., 2020
*Oryza sativa*	G1	–	–	Sukawa and Okamoto, 2018
*Triticum aestivum*	G1	–	–	Pónya et al., 1999
	–	G1	–	Bannikova et al., 1985
*Nicotiana tabacum*	G1 or S	G1	–	Tian et al., 2005
	G2	–	–	D'Amato et al., 1965
	S	–	–	Hesemann, 1973
	–	G1	–	Bannikova et al., 1985
*Lilium regale*	–	G1	–	Bannikova et al., 1985
*Lilium longiflorum*	G2	–	–	Bino et al., 1990

Discrepancies have also emerged in determining the cell cycle stage of egg and central cells, possibly due to the technical challenges associated with collecting these types of cells; female gametes develop inside the ovules, protected by layers of maternal tissues, and are difficult to isolate in sufficient numbers for standard ploidy analysis, such as flow cytometry. Consequently, the commonly used methods for assessing the ploidy and cell cycle stages of female gametes rely on DNA staining, observation of the expression dynamics of cell cycle markers and incorporation of nucleotide analogues (Barrell and Grossniklaus, 2005; Liu et al., 2020; She et al., 2013; Simonini et al., 2024; Voichek et al., 2023) (Table 1; Fig. 4). Two recent studies have explored the cell cycle stages at which *Arabidopsis* egg and central cells pause before fertilization. Voichek et al. (2023) propose that the mature egg cell and central cell are arrested in the G0 and G1 phases, respectively, without replicating their DNA before fertilization. This conclusion is supported by the absence of markers for the G1-phase, pre-replication, or mitosis, along with the diffuse fluorescence of S-phase markers such as PCNA1-GFP (which indicates a lack of DNA replication) in the mature egg cell. The authors observe a similar expression pattern in the central cell, with a general absence of most markers, except for weak expression of some G1-phase and pre-replication markers, from which they conclude that the central cell is in G1. However, Simonini et al. (2024) present an alternative view, proposing that the egg cell is in G2 phase, while the central cell is arrested in S-phase. These conclusions are supported by propidium iodide staining to quantify DNA content, EdU incorporation to detect DNA synthesis, and the use of various marker lines, in a strategy similar to that employed by Voichek and colleagues. The discrepancies between the two studies may stem from challenges in interpreting the expression profiles of the markers used. While these cell cycle markers perform well in tissues like roots, leaves and meristems (D'Ario et al., 2021; Desvoyes et al., 2020; Han et al., 2022), they often yield inconsistent or weak signals in the female gametophyte (Simonini et al., 2024; Voichek et al., 2023). This limitation highlights the need to complement marker-based analysis with additional techniques to accurately capture the dynamics in female gametes.

The molecular basis of cell cycle arrest in quiescent gametes has also been explored. Expression of the CYCB;1 marker is undetectable in mature female gametophytes awaiting fertilization, which is consistent with cell cycle arrest (Johnston et al., 2008). RBR1 plays a pivotal role in the establishment of the quiescent state by preventing divisions within the mature female gametophyte (Ebel et al., 2004; Johnston et al., 2008). Indeed, *rbr1* mutant female gametophytes exhibit supernumerary nuclei at the micropylar end, likely originating from the overproliferation of the egg apparatus (Ebel et al., 2004; Johnston et al., 2008). Moreover, *rbr1* mutant unfertilized central cells can initiate endosperm development autonomously, further indicating improper cell cycle arrest (Ebel et al., 2004; Ingouff et al., 2007; Johnston et al., 2008; Simonini et al., 2024). Thus, RBR1 functions as a brake that blocks cell cycle progression in the female gametes.

Autonomous division of unfertilized central cells can also be promoted by perturbations to factors beyond the core cell cycle components. Mutations in the four subunits of the Fertilization-Independent-Seed *Polycomb* Repressive Complex 2 (FIS-PRC2) [FERTILIZATION INDEPENDENT ENDOSPERM (FIE), FERTILIZATION INDEPENDENT SEED (FIS2), MULTICOPY SUPPRESSOR OF IRA1 (MSI1) and MEDEA (MEA) (Chaudhury et al., 1997; Grossniklaus et al., 1998; Kiyosue et al., 1999; Köhler et al., 2003; Ohad et al., 1996)] lead to autonomous division of the central cell and the formation of an endosperm-like structure. The absence of the PRC2 subunits MSI1 (in *Arabidopsis*) and FIE (in rice) triggers the formation of embryo-like structures, which originate from the autonomous division of the unfertilized egg cell (Guitton and Berger, 2005; Köhler et al., 2003; Wu et al., 2023). The molecular mechanism by which PRC2 regulates cell cycle arrest in female gametes remains largely unknown. It is hypothesized that PRC2 may directly regulate the gene expression of core cell cycle components, such as A- and D-type CYCs (Adhikari and Davie, 2020; Iovino et al., 2013; Johnston et al., 2008; Simonini et al., 2021). Additionally, PRC2 might interact directly with RBR1 through MSI1 (Ach et al., 1997b; Mosquna et al., 2004; Jullien et al., 2008), further influencing cell cycle control.

Despite these recent findings, much remains to be understood about cell cycle regulation in plant gametes. What mechanisms trigger cell cycle arrest in S-phase in the central cell? How do egg cells and central cells maintain their quiescent state over time? New technologies that require little input material, such single-cell technologies (Baysoy et al., 2023), could eventually help resolve some of these longstanding questions and offer a benchmark approach for studying cell cycle progression in plant gametes more comprehensively.

## Cell cycle reactivation in the gametes at the moment of fertilization

In vertebrates, fully grown oocytes are arrested at the prophase of the first meiosis (prophase I). In the final stage of their maturation, they prepare for fertilization: meiosis is resumed but the egg arrests again at metaphase of the second meiosis (metaphase II). Quiescency in the egg is achieved by inactivation of the APC/C complex, so that destruction of CycB is prevented and cell cycle arrest is maintained (Hörmanseder et al., 2013). At fertilization, the sperm breaks this cell cycle arrest by delivering a phospholipase C protein, which generates calcium oscillations in the fertilized egg (Madgwick et al., 2005; Madgwick and Jones, 2007; Saunders et al., 2002). This Ca^2+^ surge activates the calmodulin-dependent protein kinase II (CamKII), which in turn activates the APC/C complex (Stricker, 1999). This results in the destruction of CycB1 and cell cycle progression (Nixon et al., 2002). Ca^2+^ oscillations in the egg are sufficient to initiate the entire signalling cascade required to reinstate cell division (Machaty, 2024). Indeed, parthenogenesis can be induced by adding calcium ionophores (molecules that cause the release of intracellular Ca^2+^) to sea urchin eggs (Steinhardt and Epel, 1974).

A calcium wave also occurs during fertilization in plants. Unfertilized egg and central cells have low calcium concentrations (Denninger et al., 2014; Hamamura et al., 2014). Upon pollen tube rupture and sperm discharge, a calcium spike pervades the entire embryo sac, including the egg and central cells (Denninger et al., 2014; Digonnet et al., 1997; Hamamura et al., 2014; Ngo et al., 2014). However, unlike in animals, calcium influx is not sufficient to induce parthenogenetic embryo development in plants, suggesting that additional factors, which might be delivered by the sperm, are required to activate cell division (Koltunow and Grossniklaus, 2003).

**Fig. 5.**
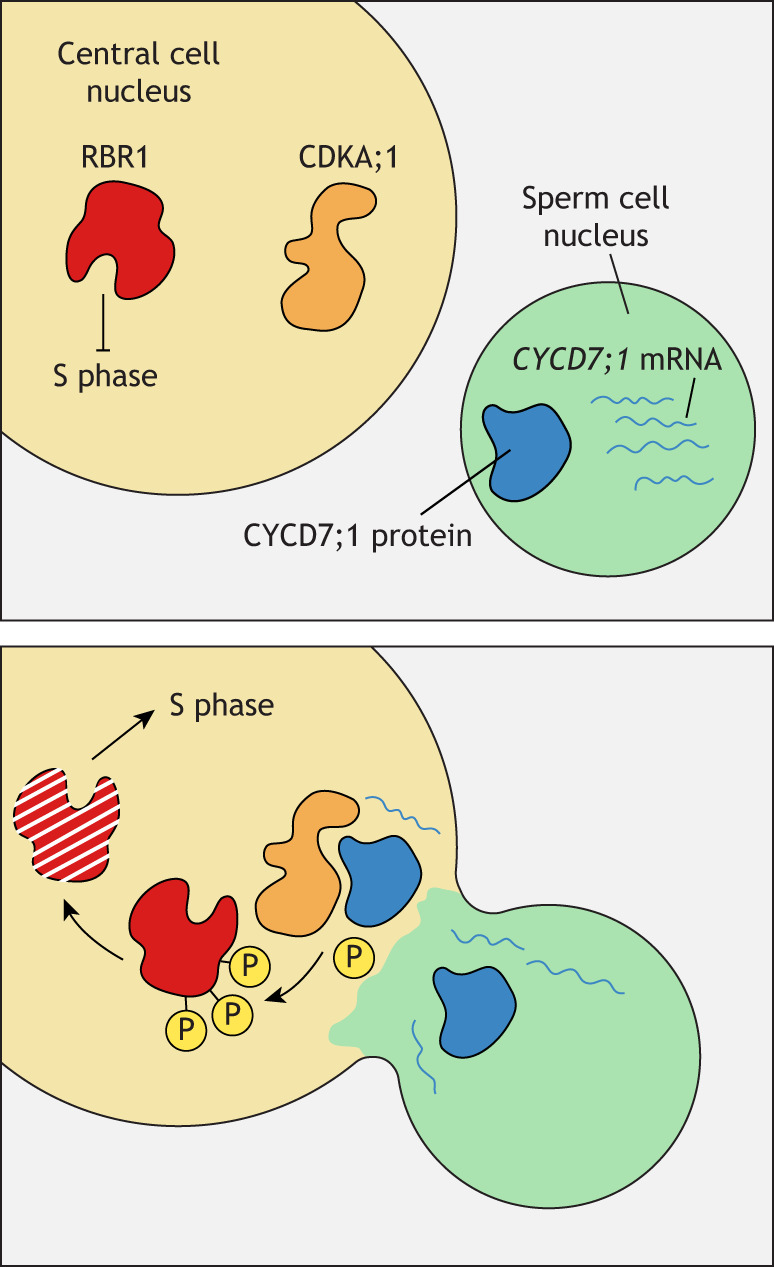
**Cell cycle reactivation in the central cell.** Cell cycle progression in the central cell is halted by RBR1 activity (top panel). During karyogamy (bottom panel), the delivery of paternally produced CYCD7;1 protein and its messenger RNA forms an active CDKA;1–CYCD complex. This complex triggers RBR1 degradation, thereby enabling cell cycle progression.

One such factor is the D-type cyclin CYCD7;1. The CYCD7;1 protein and its messenger RNA are carried in the sperm and delivered to the female gametes through karyogamy (Simonini et al., 2024). CYCD7;1 induces RBR1 degradation in the central cell, thus releasing the central cell from quiescence and allowing it to complete DNA replication (Simonini et al., 2024; Fig. 5). When a central cell receives pollen lacking CYCD7;1, RBR1 is not degraded, and so cell cycle arrest in the central cell persists (Simonini et al., 2024). This mechanism appears to be specific to the central cell, as the egg cell is not affected by absence of paternally-derived CYCD7;1, and RBR1 absence (either through mutation or induced degradation) does not stimulate egg cell cycle progression (Ebel et al., 2004; Ingouff et al., 2009; Johnston et al., 2008; Simonini et al., 2024). Thus, central cells and egg cells regulate their cell cycles differently.

Other paternally derived or paternally expressed factors have also been shown to regulate the cell cycle after fertilization. For example, paternal alleles of auxin biosynthetic genes of the YUCCA family are required for auxin production in the female gametes to induce endosperm proliferation (Figueiredo et al., 2016, 2015). Moreover, the transcription factor BABY BOOM (BBM) accumulates in the sperm, and its delivery is required to initiate embryogenesis (Khanday et al., 2023; Ren et al., 2024). Indeed, ectopic expression of BBM in plant egg cells induces autonomous proliferation of the egg and thus initiates embryogenesis (Boutilier et al., 2002; Chen et al., 2022; Conner et al., 2015; Khanday et al., 2023; Passarinho et al., 2008). The *PARTHENOGENESIS* (*PAR*) gene, identified in apomictic (asexually reproducing) dandelions, is sufficient to induce embryogenesis without fertilization when introduced into lettuce egg cells, a sexually reproducing species (Underwood et al., 2022). In sexually reproducing plants, PAR alleles are typically expressed in pollen, suggesting that the gene product acts at fertilization to lift a block on cell division (Underwood et al., 2022).

After karyogamy with the sperm nucleus, the fertilized egg cell and central cell initiate division at very different speeds. While the zygote takes about 16-20 h before committing its first division, the primary endosperm nucleus initiates mitotic divisions about 4-6 h after fertilization (Maruyama et al., 2015; Simonini et al., 2024). Mutations affecting subunits necessary for DNA replication lead to cell cycle arrest during early seed development. For example, mutations in subunits of the Origin Recognition Complex (ORC), in the Mini Chromosome Maintenance family members (MCMs) and in DNA replication licensing factors (RFCs), induce seed abortion, producing an embryo arrested at very early stages and an underdeveloped endosperm with extremely enlarged nuclei and disc-shaped or pitted nucleoli (Collinge et al., 2004; Herridge et al., 2014; Ni et al., 2009; Qian et al., 2018; Springer et al., 2000).

Cdk1 activity appears to be important for embryogenesis in animals; for example, Cdk1 knockout mice die before reaching the morula stage (Santamaría et al., 2007; Satyanarayana et al., 2008). Surprisingly, in plants, seeds that lack functional CDKA;1 can still develop fully, although they exhibit slower growth and fewer cell divisions compared to wild-type seeds (Nowack et al., 2012). Removing CDKB activity further enhances the *cdka;1* mutant phenotype, with a small percentage of seeds from *CDKA;1/cdka;1 cdkb;1/cdkb1;1* mutant plants containing embryos arrested at a range of different stages of seed development (Nowack et al., 2012).

## Conclusions

For decades – if not centuries – biologists have investigated the processes that govern gamete development, reproduction, fertilization and embryogenesis. Central to the study of these fundamental aspects of developmental biology is the need to comprehend how cells function and divide. It is now clear that cell division is not merely a mechanism for increasing cell numbers within tissues or organs. Rather, it is intricately linked to tissue patterning, cellular identity acquisition, and the establishment of body axes and polarity. This regulatory role of cell division is especially prominent during plant development. Unlike animal cells, plant cells are encased in a rigid cell wall, rendering them immobile from the earliest stages of embryogenesis. As a result, plants rely heavily on spatially and temporally regulated cell divisions – often directional or asymmetric – to initiate identity acquisition, tissue and organ patterning, and the development of body axes throughout their entire life cycle.

In addition to understanding the molecular mechanisms that control cell division, it is equally important to explore the signals that trigger cell cycle arrest or reactivation. These processes are fundamental not only for reproduction but also in broader biological contexts, such as in the study of how certain types of diseases, such as cancer, arise. In the context of plant development, identifying the signals and key players that regulate cell division is crucial for enhancing reproductive strategies to secure global food production. One promising approach is apomixis, a form of asexual reproduction where seeds form without fertilization (Goeckeritz et al., 2024; Koltunow and Grossniklaus, 2003; Ozias-Akins and Conner, 2020). Engineering crops for apomixis could significantly boost yields and reduce reliance on traditional breeding methods. To accomplish this, it is necessary to bypass the natural quiescent state of plant gametes and activate the cell division pathways that typically govern embryo and endosperm development. Therefore, understanding the molecular mechanisms controlling cell cycle arrest and reactivation during plant reproduction brings us closer to achieving major breakthroughs in plant breeding and sustainable agriculture.

Over the past few decades, technological advancements have dramatically enhanced our ability to study cell division at both the molecular and cellular levels. Since the early yeast screenings of the 1970s, many of the core factors involved in cell cycle progression have been identified. However, in the context of reproduction, particularly in plants, much of the knowledge gained remains largely descriptive, and deep knowledge about the regulation of cell division at the molecular level during plant reproduction is still missing. For example, what are the signals that induce cell cycle arrest in mature plant gametes? What triggers the reactivation of the cell cycle? In the case of the central cell, we now know that its reactivation depends on the paternal delivery of core cell cycle components, but what about the egg cell? Understanding how these distinct gametes maintain their arrested states and what mechanisms control their reactivation remains an open question in plant reproductive and developmental biology. Technological innovation will be pivotal in answering these questions. Recently developed single-cell technologies allow us to achieve cellular resolution and understand the transcriptomes of individual cells, shedding light on their status during different stages of development. However, since cell cycle progression and regulation primarily occur at the protein level, there is still the need for methods that can detect protein species, their abundance from limited sample sizes and also their associated post-translational modification, such as phosphorylation and ubiquitination. The coming years could see the development and optimization of game-changing techniques, such as spatial proteomics. This would enable us to monitor protein accumulation, degradation and interactions between cell cycle components with cellular resolution – all without the need to isolate cells from their native environment. Plants present an ideal model system for applying these cutting-edge technologies, particularly for studying cell cycle dynamics during gamete development and reproduction. Unlike animals, where gametes are formed during embryonic development, plant gametes develop at much later stages, making plants an excellent system for studying the early steps of gametogenesis in real time. Additionally, plants produce gametes in abundance, providing a significant advantage when considering sample sizes and the reproducibility of experimental results. Another key benefit is that mutations affecting cell cycle components, which often induce abortion of the embryo in animals, are viable in plants, allowing us to study their effects on reproduction and development. Even in cases where mutations prove lethal in plants, it is still possible to analyze segregating populations to assess the impact of these mutations during gametogenesis. This flexibility offers unique opportunities to dissect the role of specific cell cycle regulators and their broader implications during development. The knowledge gained from such studies will bridge the gap between purely descriptive observations and a deeper, mechanistic understanding of how cell division is regulated during the development of multicellular organisms.

## References

[DEV204217C1] Ach, R. A., Durfee, T., Miller, A. B., Taranto, P., Hanley-Bowdoin, L., Zambryski, P. C. and Gruissem, W. (1997a). RRB1 and RRB2 encode maize retinoblastoma-related proteins that interact with a plant D-type cyclin and geminivirus replication protein. *Mol. Cell. Biol.* 17, 5077-5086. 10.1128/MCB.17.9.5077 9271385 PMC232358

[DEV204217C2] Ach, R. A., Taranto, P. and Gruissem, W. (1997b). A conserved family of WD-40 proteins binds to the retinoblastoma protein in both plants and animals. *Plant Cell* 9, 1595-1606. 10.1105/tpc.9.9.1595 9338962 PMC157036

[DEV204217C3] Adhikari, A. and Davie, J. K. (2020). The PRC2 complex directly regulates the cell cycle and controls proliferation in skeletal muscle. *Cell Cycle Georget. Tex.* 19, 2373-2394. 10.1080/15384101.2020.1806448 PMC7513841 32816597

[DEV204217C4] Adhikari, D., Flohr, G., Gorre, N., Shen, Y., Yang, H., Lundin, E., Lan, Z., Gambello, M. J. and Liu, K. (2009). Disruption of Tsc2 in oocytes leads to overactivation of the entire pool of primordial follicles. *Mol. Hum. Reprod* 15, 765-770. 10.1093/molehr/gap092 19843635

[DEV204217C5] Adhikari, D., Zheng, W., Shen, Y., Gorre, N., Hämäläinen, T., Cooney, A. J., Huhtaniemi, I., Lan, Z.-J. and Liu, K. (2010). Tsc/mTORC1 signaling in oocytes governs the quiescence and activation of primordial follicles. *Hum. Mol. Genet* 19, 397-410. 10.1093/hmg/ddp483 19843540 PMC2798719

[DEV204217C6] Alvarez-Buylla, E. R., Benítez, M., Corvera-Poiré, A., Chaos Cador, A., de Folter, S., Gamboa de Buen, A., Garay-Arroyo, A., García-Ponce, B., Jaimes-Miranda, F., Pérez-Ruiz, R. V. et al. (2010). Flower development. *Arab. Book* 8, e0127. 10.1199/tab.0127 PMC3244948 22303253

[DEV204217C7] Angus, S. P., Mayhew, C. N., Solomon, D. A., Braden, W. A., Markey, M. P., Okuno, Y., Cardoso, M. C., Gilbert, D. M. and Knudsen, E. S. (2004). RB reversibly inhibits DNA replication via two temporally distinct mechanisms. *Mol. Cell. Biol.* 24, 5404-5420. 10.1128/MCB.24.12.5404-5420.2004 15169903 PMC419877

[DEV204217C8] Araki, T. (2001). Transition from vegetative to reproductive phase. *Curr. Opin. Plant Biol.* 4, 63-68. 10.1016/S1369-5266(00)00137-0 11163170

[DEV204217C9] Arellano, M. and Moreno, S. (1997). Regulation of CDK/cyclin complexes during the cell cycle. *Int. J. Biochem. Cell Biol.* 29, 559-573. 10.1016/s1357-2725(96)00178-1 9363633

[DEV204217C10] Avanzi, S. and Cionini, P. G. (1971). A DNA cytophotometric investigation on the development of the female gametophyte of ginkgo biloba. *Caryologia* 24, 105-116. 10.1080/00087114.1971.10796418

[DEV204217C11] Aw, S. J., Hamamura, Y., Chen, Z., Schnittger, A. and Berger, F. (2010). Sperm entry is sufficient to trigger division of the central cell but the paternal genome is required for endosperm development in Arabidopsis. *Development* 137, 2683-2690. 10.1242/dev.052928 20610483

[DEV204217C12] Azumi, Y., Liu, D., Zhao, D., Li, W., Wang, G., Hu, Y. and Ma, H. (2002). Homolog interaction during meiotic prophase I in Arabidopsis requires the SOLO DANCERS gene encoding a novel cyclin-like protein. *EMBO J.* 21, 3081-3095. 10.1093/emboj/cdf285 12065421 PMC126045

[DEV204217C13] Bähler, J. (2005). Cell-cycle control of gene expression in budding and fission yeast. *Annu. Rev. Genet* 39, 69-94. 10.1146/annurev.genet.39.110304.095808 16285853

[DEV204217C14] Bannikova, V. P., Khvedynich, O. A. and Shpilevaya, S. P. (1985). *Polovye kletki i oplodotvorenie u pokrytosemennykh i vodoroslei (Reproductive Cells and Fertilization in Angiosperms and Algae)*. Kiev: Nauk. Dumka.

[DEV204217C15] Barrell, P. J. and Grossniklaus, U. (2005). Confocal microscopy of whole ovules for analysis of reproductive development: the elongate1 mutant affects meiosis II. *Plant J. Cell Mol. Biol.* 43, 309-320. 10.1111/j.1365-313X.2005.02456.x 15998316

[DEV204217C16] Barrett, S. C. H. (2002). The evolution of plant sexual diversity. *Nat. Rev. Genet.* 3, 274-284. 10.1038/nrg776 11967552

[DEV204217C17] Bartoli, G., Felici, C. and Ruffini Castiglione, M. (2017). Female gametophyte and embryo development in Helleborus bocconei Ten. Ranunculaceae). *Protoplasma* 254, 491-504. 10.1007/s00709-016-0969-8 27048178

[DEV204217C18] Baysoy, A., Bai, Z., Satija, R. and Fan, R. (2023). The technological landscape and applications of single-cell multi-omics. *Nat. Rev. Mol. Cell Biol.* 24, 695-713. 10.1038/s41580-023-00615-w 37280296 PMC10242609

[DEV204217C19] Bennett, M. D. and Smith, J. B. (1976). The nuclear DNA content of the egg, the zygote and young proembryo cells in hordeum. *Caryologia* 29, 435-446. 10.1080/00087114.1976.10796517

[DEV204217C20] Berger, F. (1999). Endosperm development. *Curr. Opin. Plant Biol.* 2, 28-32. 10.1016/s1369-5266(99)80006-5 10047564

[DEV204217C21] Berger, F. and Twell, D. (2011). Germline specification and function in plants. *Annu. Rev. Plant Biol.* 62, 461-484. 10.1146/annurev-arplant-042110-103824 21332359

[DEV204217C22] Berger, F., Hamamura, Y., Ingouff, M. and Higashiyama, T. (2008). Double fertilization – caught in the act. *Trends Plant Sci.* 13, 437-443. 10.1016/j.tplants.2008.05.011 18650119

[DEV204217C23] Biedermann, S., Harashima, H., Chen, P., Heese, M., Bouyer, D., Sofroni, K. and Schnittger, A. (2017). The retinoblastoma homolog RBR 1 mediates localization of the repair protein RAD 51 to DNA lesions in Arabidopsis. *EMBO J.* 36, 1279-1297. 10.15252/embj.201694571 28320735 PMC5412766

[DEV204217C24] Bino, R. J., Tuyl, J. M. V. and De Vries, J. N. (1990). Flow cytometric determination of relative nuclear DNA contents in bicellulate and tricellulate pollen. *Ann. Bot* 65, 3-8. 10.1093/oxfordjournals.aob.a087904

[DEV204217C25] Blethrow, J. D., Glavy, J. S., Morgan, D. O. and Shokat, K. M. (2008). Covalent capture of kinase-specific phosphopeptides reveals Cdk1-cyclin B substrates. *Proc. Natl. Acad. Sci. USA* 105, 1442-1447. 10.1073/pnas.0708966105 18234856 PMC2234163

[DEV204217C26] Blilou, I., Frugier, F., Folmer, S., Serralbo, O., Willemsen, V., Wolkenfelt, H., Eloy, N. B., Ferreira, P. C. G., Weisbeek, P. and Scheres, B. (2002). The Arabidopsis HOBBIT gene encodes a CDC27 homolog that links the plant cell cycle to progression of cell differentiation. *Genes Dev.* 16, 2566-2575. 10.1101/gad.237302 12368267 PMC187454

[DEV204217C27] Borg, M., Brownfield, L. and Twell, D. (2009). Male gametophyte development: a molecular perspective. *J. Exp. Bot* 60, 1465-1478. 10.1093/jxb/ern355 19213812

[DEV204217C28] Borg, M., Buendía, D. and Berger, F. (2019). A simple and robust protocol for immunostaining Arabidopsis pollen nuclei. *Plant Reprod* 32, 39-43. 10.1007/s00497-018-00360-7 30671645

[DEV204217C29] Borges, F., Gomes, G., Gardner, R., Moreno, N., McCormick, S., Feijó, J. A. and Becker, J. D. (2008). Comparative transcriptomics of Arabidopsis sperm cells. *Plant Physiol.* 148, 1168-1181. 10.1104/pp.108.125229 18667720 PMC2556834

[DEV204217C30] Boutilier, K., Offringa, R., Sharma, V. K., Kieft, H., Ouellet, T., Zhang, L., Hattori, J., Liu, C.-M., van Lammeren, A. A. M., Miki, B. L. A. et al. (2002). Ectopic expression of BABY BOOM triggers a conversion from vegetative to embryonic growth. *Plant Cell* 14, 1737-1749. 10.1105/tpc.001941 12172019 PMC151462

[DEV204217C31] Brehm, A. and Kouzarides, T. (1999). Retinoblastoma protein meets chromatin. *Trends Biochem. Sci.* 24, 142-145. 10.1016/s0968-0004(99)01368-7 10322419

[DEV204217C32] Brewbaker, J. L. (1967). The distribution and phylogenetic significance of binucleate and trinucleate pollen grains in the angiosperms. *Am. J. Bot* 54, 1069-1083. 10.1002/j.1537-2197.1967.tb10735.x

[DEV204217C33] Brownfield, L., Hafidh, S., Borg, M., Sidorova, A., Mori, T. and Twell, D. (2009). A plant germline-specific integrator of sperm specification and cell cycle progression. *PLoS Genet.* 5, e1000430. 10.1371/journal.pgen.1000430 19300502 PMC2653642

[DEV204217C34] Bulankova, P., Akimcheva, S., Fellner, N. and Riha, K. (2013). Identification of Arabidopsis meiotic cyclins reveals functional diversification among plant cyclin genes. *PLoS Genet.* 9, e1003508. 10.1371/journal.pgen.1003508 23671425 PMC3649987

[DEV204217C35] Burke, J. R., Deshong, A. J., Pelton, J. G. and Rubin, S. M. (2010). Phosphorylation-induced conformational changes in the retinoblastoma protein inhibit E2F transactivation domain binding. *J. Biol. Chem.* 285, 16286-16293. 10.1074/jbc.M110.108167 20223825 PMC2871496

[DEV204217C36] Cairo, A., Vargova, A., Shukla, N., Capitao, C., Mikulkova, P., Valuchova, S., Pecinkova, J., Bulankova, P. and Riha, K. (2022). Meiotic exit in Arabidopsis is driven by P-body-mediated inhibition of translation. *Science* 377, 629-634. 10.1126/science.abo0904 35926014

[DEV204217C37] Cao, L., Wang, S., Venglat, P., Zhao, L., Cheng, Y., Ye, S., Qin, Y., Datla, R., Zhou, Y. and Wang, H. (2018). Arabidopsis ICK/KRP cyclin-dependent kinase inhibitors function to ensure the formation of one megaspore mother cell and one functional megaspore per ovule. *PLoS Genet.* 14, e1007230. 10.1371/journal.pgen.1007230 29513662 PMC5858843

[DEV204217C38] Capron, A. (2003). The Arabidopsis anaphase-promoting complex or cyclosome: molecular and genetic characterization of the APC2 subunit. *Plant Cell Online* 15, 2370-2382. 10.1105/tpc.013847 PMC197302 14508008

[DEV204217C39] Carmichael, J. S. and Friedman, W. E. (1995). Double fertilization in gnetum gnemon: the relationship between the cell cycle and sexual reproduction. *Plant Cell* 7, 1975-1988. 10.1105/tpc.7.12.1975 12242365 PMC161055

[DEV204217C40] Castro, A. and Lorca, T. (2018). Greatwall kinase at a glance. *J. Cell Sci.* 131, jcs222364. 10.1242/jcs.222364 30355803

[DEV204217C41] Ceccato, L., Masiero, S., Sinha Roy, D., Bencivenga, S., Roig-Villanova, I., Ditengou, F. A., Palme, K., Simon, R. and Colombo, L. (2013). Maternal control of PIN1 is required for female gametophyte development in Arabidopsis. *PLoS ONE* 8, e66148. 10.1371/journal.pone.0066148 23799075 PMC3684594

[DEV204217C42] Chahtane, H., Lai, X., Tichtinsky, G., Rieu, P., Arnoux-Courseaux, M., Cancé, C., Marondedze, C. and Parcy, F. (2023). Flower Development in Arabidopsis. In *Flower Development : Methods and Protocols* (ed. J. L. Riechmann and C. Ferrándiz), pp. 3-38. New York, NY: Springer US. 10.1007/978-1-0716-3299-4_1 37540352

[DEV204217C43] Chaudhury, A. M., Ming, L., Miller, C., Craig, S., Dennis, E. S. and Peacock, W. J. (1997). Fertilization-independent seed development in Arabidopsis thaliana. *Proc. Natl. Acad. Sci. USA* 94, 4223-4228. 10.1073/pnas.94.8.4223 9108133 PMC20611

[DEV204217C44] Chen, Z., Hafidh, S., Poh, S. H., Twell, D. and Berger, F. (2009). Proliferation and cell fate establishment during Arabidopsis male gametogenesis depends on the Retinoblastoma protein. *Proc. Natl. Acad. Sci. USA* 106, 7257-7262. 10.1073/pnas.0810992106 19359496 PMC2678419

[DEV204217C45] Chen, J., Strieder, N., Krohn, N. G., Cyprys, P., Sprunck, S., Engelmann, J. C. and Dresselhaus, T. (2017). Zygotic genome activation occurs shortly after fertilization in maize. *Plant Cell* 29, 2106-2125. 10.1105/tpc.17.00099 28814645 PMC5635985

[DEV204217C46] Chen, B., Maas, L., Figueiredo, D., Zhong, Y., Reis, R., Li, M., Horstman, A., Riksen, T., Weemen, M., Liu, H. et al. (2022). BABY BOOM regulates early embryo and endosperm development. *Proc. Natl. Acad. Sci. USA* 119, e2201761119. 10.1073/pnas.2201761119 35709319 PMC9231476

[DEV204217C347] Chudinova, E. M., Karpov, P. A., Fokin, A. I., Yemets, A. I., Lytvyn, D. I., Nadezhdina, E. S. and Blume, Y. B. (2017). MAST-like protein kinase IREH1 from Arabidopsis thaliana co-localizes with the centrosome when expressed in animal cells. *Planta* 246, 959-969. 10.1007/s00425-017-2742-4 28717875

[DEV204217C47] Churchman, M. L., Brown, M. L., Kato, N., Kirik, V., Hülskamp, M., Inzé, D., De Veylder, L., Walker, J. D., Zheng, Z., Oppenheimer, D. G. et al. (2006). SIAMESE, a plant-specific cell cycle regulator, controls endoreplication onset in Arabidopsis thaliana. *Plant Cell* 18, 3145-3157. 10.1105/tpc.106.044834 17098811 PMC1693949

[DEV204217C48] Cifuentes, M., Jolivet, S., Cromer, L., Harashima, H., Bulankova, P., Renne, C., Crismani, W., Nomura, Y., Nakagami, H., Sugimoto, K. et al. (2016). TDM1 regulation determines the number of meiotic divisions. *PLoS Genet.* 12, e1005856. 10.1371/journal.pgen.1005856 26871453 PMC4752240

[DEV204217C49] Collinge, M. A., Spillane, C., Köhler, C., Gheyselinck, J. and Grossniklaus, U. (2004). Genetic interaction of an origin recognition complex subunit and the *Polycomb* group gene *MEDEA* during seed development. *Plant Cell* 16, 1035-1046. 10.1105/tpc.019059 15020747 PMC412875

[DEV204217C50] Conner, J. A., Mookkan, M., Huo, H., Chae, K. and Ozias-Akins, P. (2015). A parthenogenesis gene of apomict origin elicits embryo formation from unfertilized eggs in a sexual plant. *Proc. Natl. Acad. Sci. USA* 112, 11205-11210. 10.1073/pnas.1505856112 26305939 PMC4568661

[DEV204217C51] d'Erfurth, I., Cromer, L., Jolivet, S., Girard, C., Horlow, C., Sun, Y., To, J. P. C., Berchowitz, L. E., Copenhaver, G. P. and Mercier, R. (2010). The cyclin-A CYCA1;2/TAM is required for the meiosis I to meiosis II transition and cooperates with OSD1 for the prophase to first meiotic division transition. *PLoS Genet.* 6, e1000989. 10.1371/journal.pgen.1000989 20585549 PMC2887465

[DEV204217C52] D'Amato, F., Devreux, M. and Mugnozza, G. T. S. (1965). The DNA content of the nuclei of the pollen grains in tobacco and barley. *Caryologia* 18, 377-382. 10.1080/00087114.1965.10796180

[DEV204217C53] D'Ario, M. and Sablowski, R. (2019). Cell size control in plants. *Annu. Rev. Genet.* 53, 45-65. 10.1146/annurev-genet-112618-043602 31430180

[DEV204217C54] D'Ario, M., Tavares, R., Schiessl, K., Desvoyes, B., Gutierrez, C., Howard, M. and Sablowski, R. (2021). Cell size controlled in plants using DNA content as an internal scale. *Science* 372, 1176-1181. 10.1126/science.abb4348 34112688

[DEV204217C55] Deng, H., Song, Y. X., Qin, K. and Tian, H. Q. (2012). DNA content and cell cycle changes of male and female gametes of *Lycium barbarum* L. *Plant Physiol. J.* 48, 869-873.

[DEV204217C56] De Schutter, K., Joubès, J., Cools, T., Verkest, A., Corellou, F., Babiychuk, E., Van Der Schueren, E., Beeckman, T., Kushnir, S., Inzé, D. et al. (2007). Arabidopsis WEE1 kinase controls cell cycle arrest in response to activation of the DNA integrity checkpoint. *Plant Cell* 19, 211-225. 10.1105/tpc.106.045047 17209125 PMC1820959

[DEV204217C57] De Veylder, L., Beeckman, T., Beemster, G. T., Krols, L., Terras, F., Landrieu, I., van der Schueren, E., Maes, S., Naudts, M. and Inzé, D. (2001). Functional analysis of cyclin-dependent kinase inhibitors of Arabidopsis. *Plant Cell* 13, 1653-1668. 10.1105/tpc.010087 11449057 PMC139548

[DEV204217C58] Denninger, P., Bleckmann, A., Lausser, A., Vogler, F., Ott, T., Ehrhardt, D. W., Frommer, W. B., Sprunck, S., Dresselhaus, T. and Grossmann, G. (2014). Male–female communication triggers calcium signatures during fertilization in Arabidopsis. *Nat. Commun.* 5, 4645. 10.1038/ncomms5645 25145880 PMC4143946

[DEV204217C59] Desnoyer, N. J. and Grossniklaus, U. (2023). Live imaging of Arabidopsis pollen tube reception and double fertilization using the semi-in vitro cum septum method. *J. Vis. Exp.*, e65156. 10.3791/65156 36912546

[DEV204217C60] Desvoyes, B., Arana-Echarri, A., Barea, M. D. and Gutierrez, C. (2020). A comprehensive fluorescent sensor for spatiotemporal cell cycle analysis in Arabidopsis. *Nat. Plants* 6, 1330-1334. 10.1038/s41477-020-00770-4 32989288

[DEV204217C61] Digonnet, C., Aldon, D., Leduc, N., Dumas, C. and Rougier, M. (1997). First evidence of a calcium transient in flowering plants at fertilization. *Development* 124, 2867-2874. 10.1242/dev.124.15.2867 9247330

[DEV204217C62] Dissmeyer, N., Nowack, M. K., Pusch, S., Stals, H., Inzé, D., Grini, P. E. and Schnittger, A. (2007). T-loop phosphorylation of Arabidopsis CDKA;1 is required for its function and can be partially substituted by an aspartate residue. *Plant Cell* 19, 972-985. 10.1105/tpc.107.050401 17369369 PMC1867360

[DEV204217C63] Dissmeyer, N., Weimer, A. K., Pusch, S., De Schutter, K., Alvim Kamei, C. L., Nowack, M. K., Novak, B., Duan, G.-L., Zhu, Y.-G., De Veylder, L. et al. (2009). Control of cell proliferation, organ growth, and DNA damage response operate independently of dephosphorylation of the Arabidopsis Cdk1 homolog CDKA;1. *Plant Cell* 21, 3641-3654. 10.1105/tpc.109.070417 19948791 PMC2798325

[DEV204217C64] Dissmeyer, N., Weimer, A. K., De Veylder, L., Novak, B. and Schnittger, A. (2010). The regulatory network of cell cycle progression is fundamentally different in plants versus yeast or metazoans. *Plant Signal. Behav.* 5, 1613-1618. 10.4161/psb.5.12.13969 21139435 PMC3115114

[DEV204217C65] Dowdy, S. F., Hinds, P. W., Louie, K., Reed, S. I., Arnold, A. and Weinberg, R. A. (1993). Physical interaction of the retinoblastoma protein with human D cyclins. *Cell* 73, 499-511. 10.1016/0092-8674(93)90137-f 8490963

[DEV204217C66] Draetta, G., Luca, F., Westendorf, J., Brizuela, L., Ruderman, J. and Beach, D. (1989). Cdc2 protein kinase is complexed with both cyclin A and B: evidence for proteolytic inactivation of MPF. *Cell* 56, 829-838. 10.1016/0092-8674(89)90687-9 2538242

[DEV204217C67] Dragoi, C.-M., Kaur, E., Barr, A. R., Tyson, J. J. and Novák, B. (2024). The oscillation of mitotic kinase governs cell cycle latches in mammalian cells. *J. Cell Sci.* 137, jcs261364. 10.1242/jcs.261364 38206091 PMC10911285

[DEV204217C68] Dresselhaus, T., Sprunck, S. and Wessel, G. M. (2016). Fertilization mechanisms in flowering plants. *Curr. Biol.* 26, R125-R139. 10.1016/j.cub.2015.12.032 26859271 PMC4934421

[DEV204217C69] Drews, G. N. and Koltunow, A. M. G. (2011). The female gametophyte. *Arab. Book* 9, e0155. 10.1199/tab.0155 PMC3268550 22303279

[DEV204217C70] Durbarry, A., Vizir, I. and Twell, D. (2005). Male germ line development in Arabidopsis. *duo pollen* mutants reveal gametophytic regulators of generative cell cycle progression. *Plant Physiol.* 137, 297-307. 10.1104/pp.104.053165 15618418 PMC548860

[DEV204217C71] Ebel, C., Mariconti, L. and Gruissem, W. (2004). Plant retinoblastoma homologues control nuclear proliferation in the female gametophyte. *Nature* 429, 776-780. 10.1038/nature02637 15201912

[DEV204217C72] Echevarría, C., Gutierrez, C. and Desvoyes, B. (2021). Tools for assessing cell-cycle progression in plants. *Plant Cell Physiol.* 62, 1231-1238. 10.1093/pcp/pcab066 34021583 PMC8579159

[DEV204217C73] Ermakov, I. P., Morozova, E. M. and Karpova, L. V. (1980). DNA content in nuclei of male gametophytes of some flowering plants. *Doklady Bot Sci.* 251, 32-33.

[DEV204217C74] Eloy, N. B., de Freitas Lima, M., Van Damme, D., Vanhaeren, H., Gonzalez, N., De Milde, L., Hemerly, A. S., Beemster, G. T. S., Inzé, D. and Ferreira, P. C. G. (2011). The APC/C subunit 10 plays an essential role in cell proliferation during leaf development. *Plant J. Cell Mol. Biol.* 68, 351-363. 10.1111/j.1365-313X.2011.04691.x 21711400

[DEV204217C75] Evans, T., Rosenthal, E. T., Youngblom, J., Distel, D. and Hunt, T. (1983). Cyclin: a protein specified by maternal mRNA in sea urchin eggs that is destroyed at each cleavage division. *Cell* 33, 389-396. 10.1016/0092-8674(83)90420-8 6134587

[DEV204217C76] Ewen, M. E., Sluss, H. K., Sherr, C. J., Matsushime, H., Kato, J. and Livingston, D. M. (1993). Functional interactions of the retinoblastoma protein with mammalian D-type cyclins. *Cell* 73, 487-497. 10.1016/0092-8674(93)90136-e 8343202

[DEV204217C77] Ferrell, J. E. (2002). Self-perpetuating states in signal transduction: positive feedback, double-negative feedback and bistability. *Curr. Opin. Cell Biol.* 14, 140-148. 10.1016/s0955-0674(02)00314-9 11891111

[DEV204217C78] Friedman, W. E. (1999). Expression of the cell cycle in sperm of Arabidopsis: implications for understanding patterns of gametogenesis and fertilization in plants and other eukaryotes. *Development* 126, 1065-1075. 10.1242/dev.126.5.1065 9927606

[DEV204217C79] Figueiredo, D. D., Batista, R. A., Roszak, P. J. and Köhler, C. (2015). Auxin production couples endosperm development to fertilization. *Nat. Plants* 1, 1-6. 10.1038/nplants.2015.184 27251719

[DEV204217C80] Figueiredo, D. D., Batista, R. A., Roszak, P. J., Hennig, L. and Köhler, C. (2016). Auxin production in the endosperm drives seed coat development in Arabidopsis. *eLife* 5, e20542. 10.7554/eLife.20542 27848912 PMC5135394

[DEV204217C81] Flores-Tornero, M. and Becker, J. D. (2023). Fifty years of sperm cell isolations: from structural to omic studies. *J. Exp. Bot.* 74, 3449-3461. 10.1093/jxb/erad117 37025026 PMC10299787

[DEV204217C82] Flemming, W. (1882). *Zellsubstanz, Kern und Zelltheilung*. Leipzig: Verlag von F. C. W. Vogel.

[DEV204217C83] Friedman, W. E., Gallup, W. N. and Williams, J. H. (2003). Female gametophyte development in kadsura: implications for schisandraceae, austrobaileyales, and the early evolution of flowering plants. *Int. J. Plant Sci.* 164, S293-S305. 10.1086/376877

[DEV204217C84] Friend, S. H., Bernards, R., Rogelj, S., Weinberg, R. A., Rapaport, J. M., Albert, D. M. and Dryja, T. P. (1986). A human DNA segment with properties of the gene that predisposes to retinoblastoma and osteosarcoma. *Nature* 323, 643-646. 10.1038/323643a0 2877398

[DEV204217C85] Fung, Y. K., Murphree, A. L., T'Ang, A., Qian, J., Hinrichs, S. H. and Benedict, W. F. (1987). Structural evidence for the authenticity of the human retinoblastoma gene. *Science* 236, 1657-1661. 10.1126/science.2885916 2885916

[DEV204217C86] García-Blanco, N., Vázquez-Bolado, A. and Moreno, S. (2019). Greatwall-endosulfine: a molecular switch that regulates PP2A/B55 protein phosphatase activity in dividing and quiescent cells. *Int. J. Mol. Sci.* 20, 6228. 10.3390/ijms20246228 31835586 PMC6941129

[DEV204217C87] Gautier, J., Minshull, J., Lohka, M., Glotzer, M., Hunt, T. and Maller, J. L. (1990). Cyclin is a component of maturation-promoting factor from Xenopus. *Cell* 60, 487-494. 10.1016/0092-8674(90)90599-a 1967981

[DEV204217C88] Gharbi-Ayachi, A., Labbé, J.-C., Burgess, A., Vigneron, S., Strub, J.-M., Brioudes, E., Van-Dorsselaer, A., Castro, A. and Lorca, T. (2010). The substrate of greatwall kinase, Arpp19, controls mitosis by inhibiting protein phosphatase 2A. *Science* 330, 1673-1677. 10.1126/science.1197048 21164014

[DEV204217C89] Glotzer, M., Murray, A. W. and Kirschner, M. W. (1991). Cyclin is degraded by the ubiquitin pathway. *Nature* 349, 132-138. 10.1038/349132a0 1846030

[DEV204217C90] Goeckeritz, C. Z., Zheng, X., Harkess, A. and Dresselhaus, T. (2024). Widespread application of apomixis in agriculture requires further study of natural apomicts. *iScience* 27, 110720. 10.1016/j.isci.2024.110720 39280618 PMC11399699

[DEV204217C91] Goff, L. J. and Coleman, A. W. (1984). Elucidation of fertilization and development in a red alga by quantitative DNA microspectrofluorometry. *Dev. Biol.* 102, 173-194. 10.1016/0012-1606(84)90183-0 6698302

[DEV204217C92] Gonzalez, N., Hernould, M., Delmas, F., Gévaudant, F., Duffe, P., Causse, M., Mouras, A. and Chevalier, C. (2004). Molecular characterization of a WEE1 gene homologue in tomato (Lycopersicon esculentum Mill.). *Plant Mol. Biol* .56, 849-861. 10.1007/s11103-004-5110-2 15821985

[DEV204217C93] Grafi, G., Burnett, R. J., Helentjaris, T., Larkins, B. A., DeCaprio, J. A., Sellers, W. R. and Kaelin, W. G. (1996). A maize cDNA encoding a member of the retinoblastoma protein family: involvement in endoreduplication. *Proc. Natl. Acad. Sci. USA* 93, 8962-8967. 10.1073/pnas.93.17.8962 8799136 PMC38577

[DEV204217C94] Griswold, M. D. (2016). Spermatogenesis: the commitment to meiosis. *Physiol. Rev.* 96, 1-17. 10.1152/physrev.00013.2015 26537427 PMC4698398

[DEV204217C95] Grossniklaus, U. and Schneitz, K. (1998). The molecular and genetic basis of ovule and megagametophyte development. *Semin. Cell Dev. Biol.* 9, 227-238. 10.1006/scdb.1997.0214 9599420

[DEV204217C96] Grossniklaus, U., Vielle-Calzada, J. P., Hoeppner, M. A. and Gagliano, W. B. (1998). Maternal control of embryogenesis by MEDEA, a polycomb group gene in Arabidopsis. *Science* 280, 446-450. 10.1126/science.280.5362.446 9545225

[DEV204217C97] Gu, X., Jiang, D., Yang, W., Jacob, Y., Michaels, S. D. and He, Y. (2011). Arabidopsis homologs of retinoblastoma-associated protein 46/48 associate with a histone deacetylase to act redundantly in chromatin silencing. *PLoS Genet.* 7, e1002366. 10.1371/journal.pgen.1002366 22102827 PMC3213158

[DEV204217C98] Guiley, K. Z., Liban, T. J., Felthousen, J. G., Ramanan, P., Litovchick, L. and Rubin, S. M. (2015). Structural mechanisms of DREAM complex assembly and regulation. *Genes Dev.* 29, 961-974. 10.1101/gad.257568.114 25917549 PMC4421984

[DEV204217C99] Guitton, A.-E. and Berger, F. (2005). Loss of function of MULTICOPY SUPPRESSOR OF IRA 1 produces nonviable parthenogenetic embryos in Arabidopsis. *Curr. Biol.* 15, 750-754. 10.1016/j.cub.2005.02.066 15854908

[DEV204217C100] Gursanscky, N., Mazurkiewicz, D., Juranić, M., Johnson, S. D., León, G., Escobar-Guzmán, R., Salinas-Gamboa, R., Amasende-Morales, I., Riboni, M., Hand, M. et al. (2020). Gene expression in isolated cowpea (Vigna unguiculata L. Walp) cells from meiosis to seed initiation. *bioRxiv*. 10.1101/2020.01.17.909945

[DEV204217C101] Gusti, A., Baumberger, N., Nowack, M., Pusch, S., Eisler, H., Potuschak, T., De Veylder, L., Schnittger, A. and Genschik, P. (2009). The Arabidopsis thaliana F-Box Protein FBL17 is essential for progression through the second mitosis during pollen development. *PLoS ONE* 4, e4780. 10.1371/journal.pone.0004780 19277118 PMC2651519

[DEV204217C102] Gutierrez, C. (2016). 25 Years of cell cycle research: what's ahead? *Trends Plant Sci.* 10, 823-833. 10.1016/j.tplants.2016.06.007 27401252

[DEV204217C103] Gutierrez, C. (2022). A journey to the core of the plant cell cycle. *Int. J. Mol. Sci.* 23, 8154. 10.3390/ijms23158154 35897730 PMC9330084

[DEV204217C104] Haga, N., Kato, K., Murase, M., Araki, S., Kubo, M., Demura, T., Suzuki, K., Müller, I., Voß, U., Jürgens, G. et al. (2007). R1R2R3-Myb proteins positively regulate cytokinesis through activation of KNOLLE transcription in Arabidopsis thaliana. *Development* 134, 1101-1110. 10.1242/dev.02801 17287251

[DEV204217C105] Hamamura, Y., Nishimaki, M., Takeuchi, H., Geitmann, A., Kurihara, D. and Higashiyama, T. (2014). Live imaging of calcium spikes during double fertilization in Arabidopsis. *Nat. Commun.* 5, 4722. 10.1038/ncomms5722 25146889 PMC4143913

[DEV204217C106] Han, S.-K., Herrmann, A., Yang, J., Iwasaki, R., Sakamoto, T., Desvoyes, B., Kimura, S., Gutierrez, C., Kim, E.-D. and Torii, K. U. (2022). Deceleration of the cell cycle underpins a switch from proliferative to terminal divisions in plant stomatal lineage. *Dev. Cell* 57, 569-582.e6. 10.1016/j.devcel.2022.01.014 35148836 PMC8926846

[DEV204217C107] Harashima, H., Shinmyo, A. and Sekine, M. (2007). Phosphorylation of threonine 161 in plant cyclin-dependent kinase A is required for cell division by activation of its associated kinase. *Plant J.* 52, 435-448. 10.1111/j.1365-313X.2007.03247.x 17764501

[DEV204217C108] Harashima, H., Dissmeyer, N. and Schnittger, A. (2013). Cell cycle control across the eukaryotic kingdom. *Trends Cell Biol.* 23, 345-356. 10.1016/j.tcb.2013.03.002 23566594

[DEV204217C109] Harbour, J. W., Luo, R. X., Santi, A. D., Postigo, A. A. and Dean, D. C. (1999). Cdk phosphorylation triggers sequential intramolecular interactions that progressively block Rb functions as cells move through G1. *Cell* 98, 859-869. 10.1016/S0092-8674(00)81519-6 10499802

[DEV204217C110] Harrison, M. M., Ceol, C. J., Lu, X. and Horvitz, H. R. (2006). Some C. elegans class B synthetic multivulva proteins encode a conserved LIN-35 Rb-containing complex distinct from a NuRD-like complex. *Proc. Natl. Acad. Sci. USA* 103, 16782-16787. 10.1073/pnas.0608461103 17075059 PMC1636532

[DEV204217C111] Harrison, M. M., Lu, X. and Horvitz, H. R. (2007). LIN-61, one of two Caenorhabditis elegans malignant-brain-tumor-repeat-containing proteins, acts with the DRM and NuRD-like protein complexes in vulval development but not in certain other biological processes. *Genetics* 176, 255-271. 10.1534/genetics.106.069633 17409073 PMC1893064

[DEV204217C112] Hartwell, L. H., Culotti, J., Pringle, J. R. and Reid, B. J. (1974). Genetic control of the cell division cycle in yeast. *Science* 183, 46-51. 10.1126/science.183.4120.46 4587263

[DEV204217C113] Hata, S. (1991). cDNA cloning of a novel cdc2+/CDC28-related protein kinase from rice. *FEBS Lett.* 279, 149-152. 10.1016/0014-5793(91)80271-4 1995335

[DEV204217C114] Hengst, L. (2004). A second RING to destroy p27Kip1. *Nat. Cell Biol.* 6, 1153-1155. 10.1038/ncb1204-1153 15573093

[DEV204217C115] Hernandez-Lagana, E., Mosca, G., Mendocilla-Sato, E., Pires, N., Frey, A., Giraldo-Fonseca, A., Michaud, C., Grossniklaus, U., Hamant, O., Godin, C. et al. (2021). Organ geometry channels reproductive cell fate in the Arabidopsis ovule primordium. *eLife* 10, e66031. 10.7554/eLife.66031 33960300 PMC8219382

[DEV204217C116] Herridge, R. P., Day, R. C. and Macknight, R. C. (2014). The role of the MCM2-7 helicase complex during Arabidopsis seed development. *Plant Mol. Biol.* 86, 69-84. 10.1007/s11103-014-0213-x 24947836

[DEV204217C117] Hesemann, C. U. (1973). Untersuchungen zur Pollenentwicklung und Pollenschlauchbildung bei hoheren Pflanzen. 111. DNS-Replikation bei vegetativen und Sperma-Kernen in reifen Pollenkornern von Gerste. *Theor. Appl. Genet.* 43, 232-241. 24425075 10.1007/BF00309139

[DEV204217C118] Hiebert, S. W., Chellappan, S. P., Horowitz, J. M. and Nevins, J. R. (1992). The interaction of RB with E2F coincides with an inhibition of the transcriptional activity of E2F. *Genes Dev.* 6, 177-185. 10.1101/gad.6.2.177 1531329

[DEV204217C119] Hisanaga, T., Yamaoka, S., Kawashima, T., Higo, A., Nakajima, K., Araki, T., Kohchi, T. and Berger, F. (2019). Building new insights in plant gametogenesis from an evolutionary perspective. *Nat. Plants* 5, 663-669. 10.1038/s41477-019-0466-0 31285561

[DEV204217C120] Hörmanseder, E., Tischer, T. and Mayer, T. U. (2013). Modulation of cell cycle control during oocyte-to-embryo transitions. *EMBO J.* 32, 2191-2203. 10.1038/emboj.2013.164 23892458 PMC3746200

[DEV204217C121] Horvath, B. M., Kourova, H., Nagy, S., Nemeth, E., Magyar, Z., Papdi, C., Ahmad, Z., Sanchez-Perez, G. F., Perilli, S., Blilou, I. et al. (2017). Arabidopsis RETINOBLASTOMA RELATED directly regulates DNA damage responses through functions beyond cell cycle control. *EMBO J.* 36, 1261-1278. 10.15252/embj.201694561 28320736 PMC5412863

[DEV204217C122] Huang, J., Zhao, L., Malik, S., Gentile, B. R., Xiong, V., Arazi, T., Owen, H. A., Friml, J. and Zhao, D. (2022). Specification of female germline by microRNA orchestrated auxin signaling in Arabidopsis. *Nat. Commun.* 13, 6960. 10.1038/s41467-022-34723-6 36379956 PMC9666636

[DEV204217C123] Ingouff, M., Jullien, P. E. and Berger, F. (2007). The female gametophyte and the endosperm control cell proliferation and differentiation of the seed coat in Arabidopsis. *Plant Cell* 18, 3491-3501. 10.1105/tpc.106.047266 PMC1785409 17172356

[DEV204217C124] Ingouff, M., Sakata, T., Li, J., Sprunck, S., Dresselhaus, T. and Berger, F. (2009). The two male gametes share equal ability to fertilize the egg cell in Arabidopsis thaliana. *Curr. Biol.* 19, R19-R20. 10.1016/j.cub.2008.11.025 19138583

[DEV204217C125] Iovino, N., Ciabrelli, F. and Cavalli, G. (2013). PRC2 controls Drosophila oocyte cell fate by repressing cell cycle genes. *Dev. Cell* 26, 431-439. 10.1016/j.devcel.2013.06.021 23932903

[DEV204217C126] Iwakawa, H., Shinmyo, A. and Sekine, M. (2006). Arabidopsis CDKA;1, a cdc2 homologue, controls proliferation of generative cells in male gametogenesis. *Plant J. Cell Mol. Biol.* 45, 819-831. 10.1111/j.1365-313X.2005.02643.x 16460514

[DEV204217C127] Johnston, A. J., Matveeva, E., Kirioukhova, O., Grossniklaus, U. and Gruissem, W. (2008). A dynamic reciprocal RBR-PRC2 regulatory circuit controls Arabidopsis gametophyte development. *Curr. Biol.* 18, 1680-1686. 10.1016/j.cub.2008.09.026 18976913

[DEV204217C128] Joubès, J., Chevalier, C., Dudits, D., Heberle-Bors, E., Inzé, D., Umeda, M. and Renaudin, J. P. (2000). CDK-related protein kinases in plants. *Plant Mol. Biol.* 43, 607-620. 10.1023/a:1006470301554 11089864

[DEV204217C129] Jullien, P. E., Mosquna, A., Ingouff, M., Sakata, T., Ohad, N. and Berger, F. (2008). Retinoblastoma and its binding partner MSI1 control imprinting in Arabidopsis. *PLoS Biol.* 6, e194. 10.1371/journal.pbio.0060194 18700816 PMC2504488

[DEV204217C331] Karpov, P. A., Nadezhdina, E. S., Yemets, A. I., Matusov, V. G., Nyporko, A. Y., Shashina, N. Y. and Blume, Y. B. (2010). Bioinformatic search of plant microtubule-and cell cycle related serine-threonine protein kinases. *BMC Genomics* 11, Suppl 1(Suppl 1):S14. 10.1186/1471-2164-11-S1-S14 PMC2822528 20158871

[DEV204217C131] Kato, J., Matsushime, H., Hiebert, S. W., Ewen, M. E. and Sherr, C. J. (1993). Direct binding of cyclin D to the retinoblastoma gene product (pRb) and pRb phosphorylation by the cyclin D-dependent kinase CDK4. *Genes Dev.* 7, 331-342. 10.1101/gad.7.3.331 8449399

[DEV204217C132] Khanday, I., Santos-Medellín, C. and Sundaresan, V. (2023). Somatic embryo initiation by rice BABY BOOM1 involves activation of zygote-expressed auxin biosynthesis genes. *New Phytol.* 238, 673-687. 10.1111/nph.18774 36707918

[DEV204217C133] Kim, H. J., Oh, S. A., Brownfield, L., Hong, S. H., Ryu, H., Hwang, I., Twell, D. and Nam, H. G. (2008). Control of plant germline proliferation by SCF(FBL17) degradation of cell cycle inhibitors. *Nature* 455, 1134-1137. 10.1038/nature07289 18948957

[DEV204217C134] Kiyosue, T., Ohad, N., Yadegari, R., Hannon, M., Dinneny, J., Wells, D., Katz, A., Margossian, L., Harada, J. J., Goldberg, R. B. et al. (1999). Control of fertilization-independent endosperm development by the *MEDEA* polycomb gene in *Arabidopsis*. *Proc. Natl. Acad. Sci. USA* 96, 4186-4191. 10.1073/pnas.96.7.4186 10097185 PMC22442

[DEV204217C135] Knudsen, E. S., Buckmaster, C., Chen, T. T., Feramisco, J. R. and Wang, J. Y. (1998). Inhibition of DNA synthesis by RB: effects on G1/S transition and S-phase progression. *Genes Dev.* 12, 2278-2292. 10.1101/gad.12.15.2278 9694794 PMC317048

[DEV204217C136] Knudsen, K. E., Booth, D., Naderi, S., Sever-Chroneos, Z., Fribourg, A. F., Hunton, I. C., Feramisco, J. R., Wang, J. Y. J. and Knudsen, E. S. (2000). RB-dependent S-phase response to DNA damage. *Mol. Cell. Biol.* 20, 7751-7763. 10.1128/MCB.20.20.7751-7763.2000 11003670 PMC86358

[DEV204217C137] Kobayashi, K., Suzuki, T., Iwata, E., Nakamichi, N., Suzuki, T., Chen, P., Ohtani, M., Ishida, T., Hosoya, H., Müller, S. et al. (2015). Transcriptional repression by MYB3R proteins regulates plant organ growth. *EMBO J.* 34, 1992-2007. 10.15252/embj.201490899 26069325 PMC4551348

[DEV204217C138] Köhler, C., Hennig, L., Bouveret, R., Gheyselinck, J., Grossniklaus, U. and Gruissem, W. (2003). Arabidopsis MSI1 is a component of the MEA/FIE Polycomb group complex and required for seed development. *EMBO J.* 22, 4804-4814. 10.1093/emboj/cdg444 12970192 PMC212713

[DEV204217C139] Koltunow, A. M. and Grossniklaus, U. (2003). Apomixis: a developmental perspective. *Annu. Rev. Plant Biol.* 54, 547-574. 10.1146/annurev.arplant.54.110901.160842 14503003

[DEV204217C140] Kong, L. J., Orozco, B. M., Roe, J. L., Nagar, S., Ou, S., Feiler, H. S., Durfee, T., Miller, A. B., Gruissem, W., Robertson, D. et al. (2000). A geminivirus replication protein interacts with the retinoblastoma protein through a novel domain to determine symptoms and tissue specificity of infection in plants. *EMBO J.* 19, 3485-3495. 10.1093/emboj/19.13.3485 10880461 PMC313951

[DEV204217C141] Korenjak, M., Taylor-Harding, B., Binné, U. K., Satterlee, J. S., Stevaux, O., Aasland, R., White-Cooper, H., Dyson, N. and Brehm, A. (2004). Native E2F/RBF complexes contain Myb-interacting proteins and repress transcription of developmentally controlled E2F target genes. *Cell* 119, 181-193. 10.1016/j.cell.2004.09.034 15479636

[DEV204217C142] Kwee, H.-S. and Sundaresan, V. (2003). The NOMEGA gene required for female gametophyte development encodes the putative APC6/CDC16 component of the Anaphase Promoting Complex in Arabidopsis. *Plant J. Cell Mol. Biol.* 36, 853-866. 10.1046/j.1365-313x.2003.01925.x 14675450

[DEV204217C143] Labbé, J. C., Capony, J. P., Caput, D., Cavadore, J. C., Derancourt, J., Kaghad, M., Lelias, J. M., Picard, A. and Dorée, M. (1989). MPF from starfish oocytes at first meiotic metaphase is a heterodimer containing one molecule of cdc2 and one molecule of cyclin B. *EMBO J.* 8, 3053-3058. 10.1002/j.1460-2075.1989.tb08456.x 2531073 PMC401383

[DEV204217C144] Latorre, I., Chesney, M. A., Garrigues, J. M., Stempor, P., Appert, A., Francesconi, M., Strome, S. and Ahringer, J. (2015). The DREAM complex promotes gene body H2A.Z for target repression. *Genes Dev.* 29, 495-500. 10.1101/gad.255810.114 25737279 PMC4358402

[DEV204217C145] Lee, W. H., Bookstein, R., Hong, F., Young, L. J., Shew, J. Y. and Lee, E. Y. (1987). Human retinoblastoma susceptibility gene: cloning, identification, and sequence. *Science* 235, 1394-1399. 10.1126/science.3823889 3823889

[DEV204217C146] Lewis, P. W., Beall, E. L., Fleischer, T. C., Georlette, D., Link, A. J. and Botchan, M. R. (2004). Identification of a Drosophila Myb-E2F2/RBF transcriptional repressor complex. *Genes Dev.* 18, 2929-2940. 10.1101/gad.1255204 15545624 PMC534653

[DEV204217C147] Litovchick, L., Sadasivam, S., Florens, L., Zhu, X., Swanson, S. K., Velmurugan, S., Chen, R., Washburn, M. P., Liu, X. S. and DeCaprio, J. A. (2007). Evolutionarily conserved multisubunit RBL2/p130 and E2F4 protein complex represses human cell cycle-dependent genes in quiescence. *Mol. Cell* 26, 539-551. 10.1016/j.molcel.2007.04.015 17531812

[DEV204217C148] Liu, J., Zhang, Y., Qin, G., Tsuge, T., Sakaguchi, N., Luo, G., Sun, K., Shi, D., Aki, S., Zheng, N. et al. (2008). Targeted degradation of the cyclin-dependent kinase inhibitor ICK4/KRP6 by RING-type E3 ligases is essential for mitotic cell cycle progression during Arabidopsis gametogenesis. *Plant Cell* 20, 1538-1554. 10.1105/tpc.108.059741 18552199 PMC2483368

[DEV204217C149] Liu, X.-Q., Shi, J.-J., Fan, H., Jiao, J., Gao, L., Tan, L., Nagawa, S. and Wang, D.-Y. (2020). Nuclear DNA replicates during zygote development in Arabidopsis and Torenia fournieri. *Plant Physiol.* 185, 137-145. 10.1093/plphys/kiaa014 PMC8133679 33631800

[DEV204217C150] Liu, Y., Deng, Y., Li, G. and Zhao, J. (2013). Replication factor C1 (RFC1) is required for double-strand break repair during meiotic homologous recombination in Arabidopsis. *Plant J. Cell Mol. Biol.* 73, 154-165. 10.1111/tpj.12024 22974522

[DEV204217C151] Lui, H., Wang, H., Delong, C., Fowke, L. C., Crosby, W. L. and Fobert, P. R. (2000). The Arabidopsis Cdc2a-interacting protein ICK2 is structurally related to ICK1 and is a potent inhibitor of cyclin-dependent kinase activity in vitro. *Plant J. Cell Mol. Biol.* 21, 379-385. 10.1046/j.1365-313x.2000.00688.x 10758489

[DEV204217C152] Machaty, Z. (2024). The signal that stimulates mammalian embryo development. *Front. Cell Dev. Biol.* 12, 1474009. 10.3389/fcell.2024.1474009 39355121 PMC11442298

[DEV204217C153] Madgwick, S. and Jones, K. T. (2007). How eggs arrest at metaphase II: MPF stabilisation plus APC/C inhibition equals Cytostatic Factor. *Cell Div.* 2, 4. 10.1186/1747-1028-2-4 17257429 PMC1794241

[DEV204217C154] Madgwick, S., Levasseur, M. and Jones, K. T. (2005). Calmodulin-dependent protein kinase II, and not protein kinase C, is sufficient for triggering cell-cycle resumption in mammalian eggs. *J. Cell Sci.* 118, 3849-3859. 10.1242/jcs.02506 16091425

[DEV204217C155] Matsuda, Y., Saito, T., Koseki, M. and Shimada, T. (1990). The Chlamydomonas non-synchronous and synchronous gametogenesis as analyzed by the activities of cell body agglutinin and cell wall lytic enzyme. *Plant Physiol.* 9, 1-6. 10.1105/tpc.016659

[DEV204217C156] Magyar, Z., Bögre, L. and Ito, M. (2016). DREAMs make plant cells to cycle or to become quiescent. *Curr. Opin. Plant Biol.* 34, 100-106. 10.1016/j.pbi.2016.10.002 27816815

[DEV204217C157] Maruyama, D., Völz, R., Takeuchi, H., Mori, T., Igawa, T., Kurihara, D., Kawashima, T., Ueda, M., Ito, M., Umeda, M. et al. (2015). Rapid elimination of the persistent synergid through a cell fusion mechanism. *Cell* 161, 907-918. 10.1016/j.cell.2015.03.018 25913191

[DEV204217C158] Masui, Y. and Markert, C. L. (1971). Cytoplasmic control of nuclear behavior during meiotic maturation of frog oocytes. *J. Exp. Zool.* 177, 129-145. 10.1002/jez.1401770202 5106340

[DEV204217C159] McCormick, S. (2004). Control of male gametophyte development. *Plant Cell* 16 Suppl., S142-S153. 10.1105/tpc.016659 15037731 PMC2643393

[DEV204217C160] McCully, M. E. (1968). Histological studies on the genus Fucus. II. Histology of the reproductive tissues. *Protoplasma* 66, 205-230.

[DEV204217C161] McGowan, C. H. and Russell, P. (1993). Human Wee1 kinase inhibits cell division by phosphorylating p34cdc2 exclusively on Tyr15. *EMBO J.* 12, 75-85. 10.1002/j.1460-2075.1993.tb05633.x 8428596 PMC413177

[DEV204217C162] Menges, M., De Jager, S. M., Gruissem, W. and Murray, J. A. H. (2005). Global analysis of the core cell cycle regulators of Arabidopsis identifies novel genes, reveals multiple and highly specific profiles of expression and provides a coherent model for plant cell cycle control: Global analysis of plant core cell cycle regulators. *Plant J.* 41, 546-566. 10.1111/j.1365-313X.2004.02319.x 15686519

[DEV204217C163] Mittnacht, S., Lees, J. A., Desai, D., Harlow, E., Morgan, D. O. and Weinberg, R. A. (1994). Distinct sub-populations of the retinoblastoma protein show a distinct pattern of phosphorylation. *EMBO J.* 13, 118-127. 10.1002/j.1460-2075.1994.tb06241.x 8306955 PMC394785

[DEV204217C164] Mittnacht, S. and Weinberg, R. A. (1991). G1/S phosphorylation of the retinoblastoma protein is associated with an altered affinity for the nuclear compartment. *Cell* 65, 381-393. 10.1016/0092-8674(91)90456-9 2018973

[DEV204217C165] Mochida, S., Maslen, S. L., Skehel, M. and Hunt, T. (2010). Greatwall phosphorylates an inhibitor of protein phosphatase 2Α that is essential for mitosis. *Science* 330, 1670-1673. 10.1126/science.1195689 21164013

[DEV204217C166] Mogensen, H. L. and Holm, P. B. (1995). Dynamics of nuclear DNA quantities during zygote development in barley. *Plant Cell* 7, 487-494. 10.1105/tpc.7.4.487 12242375 PMC160798

[DEV204217C167] Mogensen, H. L., Leduc, N., Matthys-Rochon, E. and Dumas, C. (1995). Nuclear DNA amounts in the egg and zygote of maize (Zea Mays L). *Planta* 197, 641-645. 10.1007/BF00191572

[DEV204217C168] Morozova, E. M. (2002). Additional nuclear DNA in cells of embryo sacs of Haemanthus albiflos and ornithogalum caudatum. *Biol. Bull. Russ. Acad. Sci.* 29, 192-195. 10.1023/A:1014371403332

[DEV204217C169] Morozova, E. M. and Ermakov, I. P. (1993). Cell cycle during development of male and gametophytes in angiosperms, *Fiziol**.* *Biokhim. Kul't. Rast.* 25, 297-302.

[DEV204217C170] Mosquna, A., Katz, A., Shochat, S., Grafi, G. and Ohad, N. (2004). Interaction of FIE, a polycomb protein, with pRb: a possible mechanism regulating endosperm development. *Mol. Genet. Genomics* 271, 651-657. 10.1007/s00438-004-1024-6 15221456

[DEV204217C171] Nakagami, H., Sekine, M., Murakami, H. and Shinmyo, A. (1999). Tobacco retinoblastoma-related protein phosphorylated by a distinct cyclin-dependent kinase complex with Cdc2/cyclin D in vitro. *Plant J. Cell Mol. Biol.* 18, 243-252. 10.1046/j.1365-313x.1999.00449.x 10377991

[DEV204217C172] Ngo, Q. A., Vogler, H., Lituiev, D. S., Nestorova, A. and Grossniklaus, U. (2014). A calcium dialog mediated by the FERONIA signal transduction pathway controls plant sperm delivery. *Dev. Cell* 29, 491-500. 10.1016/j.devcel.2014.04.008 24814317

[DEV204217C173] Ni, D. A., Sozzani, R., Blanchet, S., Domenichini, S., Reuzeau, C., Cella, R., Bergounioux, C. and Raynaud, C. (2009). The Arabidopsis *MCM2* gene is essential to embryo development and its over–expression alters root meristem function. *New Phytol.* 184, 311-322. 10.1111/j.1469-8137.2009.02961.x 19650778

[DEV204217C174] Nigg, E. A. (1995). Cyclin-dependent protein kinases: key regulators of the eukaryotic cell cycle. *BioEssays* 17, 471-480. 10.1002/bies.950170603 7575488

[DEV204217C175] Nixon, V. L., Levasseur, M., McDougall, A. and Jones, K. T. (2002). Ca(2+) oscillations promote APC/C-dependent cyclin B1 degradation during metaphase arrest and completion of meiosis in fertilizing mouse eggs. *Curr. Biol.* 12, 746-750. 10.1016/s0960-9822(02)00811-4 12007419

[DEV204217C176] Noir, S., Marrocco, K., Masoud, K., Thomann, A., Gusti, A., Bitrian, M., Schnittger, A. and Genschik, P. (2015). The control of Arabidopsis thaliana growth by cell proliferation and endoreplication requires the F-Box protein FBL17. *Plant Cell* 27, 1461-1476. 10.1105/tpc.114.135301 25944099 PMC4456641

[DEV204217C177] Nowack, M. K., Grini, P. E., Jakoby, M. J., Lafos, M., Koncz, C. and Schnittger, A. (2006). A positive signal from the fertilization of the egg cell sets off endosperm proliferation in angiosperm embryogenesis. *Nat. Genet.* 38, 63-67. 10.1038/ng1694 16311592

[DEV204217C178] Nowack, M. K., Harashima, H., Dissmeyer, N., Zhao, X., Bouyer, D., Weimer, A. K., De Winter, F., Yang, F. and Schnittger, A. (2012). Genetic framework of cyclin-dependent kinase function in Arabidopsis. *Dev. Cell* 22, 1030-1040. 10.1016/j.devcel.2012.02.015 22595674

[DEV204217C179] Nurse, P., Thuriaux, P. and Nasmyth, K. (1976). Genetic control of the cell division cycle in the fission yeast Schizosaccharomyces pombe. *Mol. Gen. Genet.* 146, 167-178. 10.1007/BF00268085 958201

[DEV204217C180] Oakenfull, E. A., Riou-Khamlichi, C. and Murray, J. A. H. (2002). Plant D-type cyclins and the control of G1 progression. *Philos. Trans. R. Soc. B Biol. Sci.* 357, 749-760. 10.1098/rstb.2002.1085 PMC1692988 12079670

[DEV204217C181] Ohad, N., Margossian, L., Hsu, Y. C., Williams, C., Repetti, P. and Fischer, R. L. (1996). A mutation that allows endosperm development without fertilization. *Proc. Natl. Acad. Sci. USA* 93, 5319-5324. 10.1073/pnas.93.11.5319 11607683 PMC39243

[DEV204217C182] Ötvös, K., Miskolczi, P., Marhavý, P., Cruz-Ramírez, A., Benková, E., Robert, S. and Bakó, L. (2021). Pickle recruits retinoblastoma related 1 to control lateral root formation in Arabidopsis. *Int. J. Mol. Sci.* 22, 3862. 10.3390/ijms22083862 33917959 PMC8068362

[DEV204217C183] Ozias-Akins, P. and Conner, J. A. (2020). Clonal reproduction through seeds in sight for crops. *Trends Genet.* 36, 215-226. 10.1016/j.tig.2019.12.006 31973878

[DEV204217C184] Pan, T., Gao, S., Cui, X., Wang, L. and Yan, S. (2023). APC/CCDC20 targets SCFFBL17 to activate replication stress responses in Arabidopsis. *Plant Cell* 35, 910-923. 10.1093/plcell/koac360 36503931 PMC9940874

[DEV204217C185] Pan, T., Qin, Q., Nong, C., Gao, S., Wang, L., Cai, B., Zhang, M., Wu, C., Chen, H., Li, T. et al. (2021). A novel WEE1 pathway for replication stress responses. *Nat. Plants* 7, 209-218. 10.1038/s41477-021-00855-8 33574575

[DEV204217C186] Pandey, S., Moradi, A. B., Dovzhenko, O., Touraev, A., Palme, K. and Welsch, R. (2022). Molecular control of sporophyte-gametophyte ontogeny and transition in plants. *Front. Plant Sci.* 12, 789789. 10.3389/fpls.2021.789789 35095963 PMC8793881

[DEV204217C187] Parker, L. L. and Piwnica-Worms, H. (1992). Inactivation of the p34cdc2-cyclin B complex by the human WEE1 tyrosine kinase. *Science* 257, 1955-1957. 10.1126/science.1384126 1384126

[DEV204217C188] Passarinho, P., Ketelaar, T., Xing, M., van Arkel, J., Maliepaard, C., Hendriks, M. W., Joosen, R., Lammers, M., Herdies, L., den Boer, B. et al. (2008). BABY BOOM target genes provide diverse entry points into cell proliferation and cell growth pathways. *Plant Mol. Biol.* 68, 225-237. 10.1007/s11103-008-9364-y 18663586

[DEV204217C189] Pei, Z., Deng, K., Xu, C. and Zhang, S. (2023). The molecular regulatory mechanisms of meiotic arrest and resumption in Oocyte development and maturation. *Reprod. Biol. Endocrinol.* 21, 90. 10.1186/s12958-023-01143-0 37784186 PMC10544615

[DEV204217C190] Peres, A., Churchman, M. L., Hariharan, S., Himanen, K., Verkest, A., Vandepoele, K., Magyar, Z., Hatzfeld, Y., Van Der Schueren, E., Beemster, G. T. S. et al. (2007). Novel plant-specific cyclin-dependent kinase inhibitors induced by biotic and abiotic stresses. *J. Biol. Chem.* 282, 25588-25596. 10.1074/jbc.M703326200 17599908

[DEV204217C191] Pérez-Pérez, J. M., Serralbo, O., Vanstraelen, M., González, C., Criqui, M.-C., Genschik, P., Kondorosi, E. and Scheres, B. (2008). Specialization of CDC27 function in the Arabidopsis thaliana anaphase-promoting complex (APC/C). *Plant J. Cell Mol. Biol.* 53, 78-89. 10.1111/j.1365-313X.2007.03312.x 17944809

[DEV204217C192] Pesin, J. A. and Orr-Weaver, T. L. (2008). Regulation of APC/C activators in mitosis and meiosis. *Annu. Rev. Cell Dev. Biol.* 24, 475-499. 10.1146/annurev.cellbio.041408.115949 18598214 PMC4070676

[DEV204217C193] Pilkinton, M., Sandoval, R. and Colamonici, O. R. (2007). Mammalian Mip/LIN-9 interacts with either the p107, p130/E2F4 repressor complex or B-Myb in a cell cycle-phase-dependent context distinct from the Drosophila dREAM complex. *Oncogene* 26, 7535-7543. 10.1038/sj.onc.1210562 17563750

[DEV204217C194] Pina, C., Pinto, F., Feijó, J. A. and Becker, J. D. (2005). Gene family analysis of the Arabidopsis pollen transcriptome reveals biological implications for cell growth, division control, and gene expression regulation. *Plant Physiol.* 138, 744-756. 10.1104/pp.104.057935 15908605 PMC1150393

[DEV204217C195] Pines, J. (2011). Cubism and the cell cycle: the many faces of the APC/C. *Nat. Rev. Mol. Cell Biol.* 12, 427-438. 10.1038/nrm3132 21633387

[DEV204217C196] Pomerening, J. R., Sontag, E. D. and Ferrell, J. E. (2003). Building a cell cycle oscillator: hysteresis and bistability in the activation of Cdc2. *Nat. Cell Biol.* 5, 346-351. 10.1038/ncb954 12629549

[DEV204217C197] Pónya, Z., Finy, P., Feher, A., Mitykó, J., Dudits, D. and Barnabás, B. (1999). Optimisation of introducing foreign genes into egg cells and zygotes of wheat (*Triticum aestivum L*.) via microinjection. *Protoplasma* 208, 163-172.

[DEV204217C198] Qian, J., Chen, Y., Hu, Y., Deng, Y., Liu, Y., Li, G., Zou, W. and Zhao, J. (2018). Arabidopsis replication factor C4 is critical for DNA replication during the mitotic cell cycle. *Plant J.* 94, 288-303. 10.1111/tpj.13855 29406597

[DEV204217C199] Ramírez-Parra, E., Xie, Q., Boniotti, M. B. and Gutierrez, C. (1999). The cloning of plant E2F, a retinoblastoma-binding protein, reveals unique and conserved features with animal G(1)/S regulators. *Nucleic Acids Res.* 27, 3527-3533. 10.1093/nar/27.17.3527 10446243 PMC148597

[DEV204217C200] Reddy, P., Zheng, W. and Liu, K. (2010). Mechanisms maintaining the dormancy and survival of mammalian primordial follicles. *Trends Endocrinol. Metab.* 21, 96-103. 10.1016/j.tem.2009.10.001 19913438

[DEV204217C201] Ren, H., Santner, A., del Pozo, J. C., Murray, J. A. H. and Estelle, M. (2008). Degradation of the cyclin-dependent kinase inhibitor KRP1 is regulated by two different ubiquitin E3 ligases. *Plant J. Cell Mol. Biol.* 53, 705-716. 10.1111/j.1365-313X.2007.03370.x 18005227

[DEV204217C202] Ren, H., Shankle, K., Cho, M.-J., Tjahjadi, M., Khanday, I. and Sundaresan, V. (2024). Synergistic induction of fertilization-independent embryogenesis in rice egg cells by paternal-genome-expressed transcription factors. *Nat. Plants* 10, 1892-1899. 10.1038/s41477-024-01848-z 39533074

[DEV204217C203] Romeiro Motta, M., Zhao, X., Pastuglia, M., Belcram, K., Roodbarkelari, F., Komaki, M., Harashima, H., Komaki, S., Kumar, M., Bulankova, P. et al. (2022). B1-type cyclins control microtubule organization during cell division in Arabidopsis. *EMBO Rep.* 23, e53995. 10.15252/embr.202153995 34882930 PMC8728612

[DEV204217C204] Rossi, V., Locatelli, S., Lanzanova, C., Boniotti, M. B., Varotto, S., Pipal, A., Goralik-Schramel, M., Lusser, A., Gatz, C., Gutierrez, C. et al. (2003). A maize histone deacetylase and retinoblastoma-related protein physically interact and cooperate in repressing gene transcription. *Plant Mol. Biol.* 51, 401-413. 10.1023/a:1022090916446 12602870

[DEV204217C205] Rotman, N., Durbarry, A., Wardle, A., Yang, W. C., Chaboud, A., Faure, J.-E., Berger, F. and Twell, D. (2005). A novel class of MYB factors controls sperm-cell formation in plants. *Curr. Biol.* 15, 244-248. 10.1016/j.cub.2005.01.013 15694308

[DEV204217C206] Rubin, S. M., Gall, A.-L., Zheng, N. and Pavletich, N. P. (2005). Structure of the Rb C-terminal domain bound to E2F1-DP1: a mechanism for phosphorylation-induced E2F release. *Cell* 123, 1093-1106. 10.1016/j.cell.2005.09.044 16360038

[DEV204217C207] Sablowski, R. and Gutierrez, C. (2022). Cycling in a crowd: Coordination of plant cell division, growth, and cell fate. *Plant Cell* 34, 193-208. 10.1093/plcell/koab222 34498091 PMC8774096

[DEV204217C208] Sadasivam, S. and DeCaprio, J. A. (2013). The DREAM complex: master coordinator of cell cycle-dependent gene expression. *Nat. Rev. Cancer* 13, 585-595. 10.1038/nrc3556 23842645 PMC3986830

[DEV204217C209] Saffman, E. E. and Lasko, P. (1999). Germline development in vertebrates and invertebrates. *Cell. Mol. Life Sci.* 55, 1141-1163. 10.1007/s000180050363 10442094 PMC11147032

[DEV204217C210] Santamaría, D., Barrière, C., Cerqueira, A., Hunt, S., Tardy, C., Newton, K., Cáceres, J. F., Dubus, P., Malumbres, M. and Barbacid, M. (2007). Cdk1 is sufficient to drive the mammalian cell cycle. *Nature* 448, 811-815. 10.1038/nature06046 17700700

[DEV204217C211] Satyanarayana, A., Berthet, C., Lopez-Molina, J., Coppola, V., Tessarollo, L. and Kaldis, P. (2008). Genetic substitution of Cdk1 by Cdk2 leads to embryonic lethality and loss of meiotic function of Cdk2. *Development* 135, 3389-3400. 10.1242/dev.024919 18787066 PMC2668819

[DEV204217C212] Saunders, C. M., Larman, M. G., Parrington, J., Cox, L. J., Royse, J., Blayney, L. M., Swann, K. and Lai, F. A. (2002). PLC zeta: a sperm-specific trigger of Ca(2+) oscillations in eggs and embryo development. *Development* 129, 3533-3544. 10.1242/dev.129.15.3533 12117804

[DEV204217C213] Saze, H. and Kakutani, T. (2007). Heritable epigenetic mutation of a transposon-flanked Arabidopsis gene due to lack of the chromatin-remodeling factor DDM1. *EMBO J.* 26, 3641-3652. 10.1038/sj.emboj.7601788 17627280 PMC1949009

[DEV204217C214] Schmit, F., Korenjak, M., Mannefeld, M., Schmitt, K., Franke, C., von Eyss, B., Gagrica, S., Hänel, F., Brehm, A. and Gaubatz, S. (2007). LINC, a human complex that is related to pRB-containing complexes in invertebrates regulates the expression of G2/M genes. *Cell Cycle Georget. Tex.* 6, 1903-1913. 10.4161/cc.6.15.4512 17671431

[DEV204217C215] Schmoller, K. M., Turner, J. J., Kõivomägi, M. and Skotheim, J. M. (2015). Dilution of the cell cycle inhibitor Whi5 controls budding-yeast cell size. *Nature* 526, 268-272. 10.1038/nature14908 26390151 PMC4600446

[DEV204217C216] Schneitz, K., Hülskamp, M. and Pruitt, R. E. (1995). Wild-type ovule development in Arabidopsis thaliana: a light microscope study of cleared whole-mount tissue. *Plant J.* 7, 731-749. 10.1046/j.1365-313X.1995.07050731.x

[DEV204217C217] Sekine, M., Ito, M., Uemukai, K., Maeda, Y., Nakagami, H. and Shinmyo, A. (1999). Isolation and characterization of the E2F-like gene in plants. *FEBS Lett.* 460, 117-122. 10.1016/s0014-5793(99)01296-x 10571072

[DEV204217C218] Sha, W., Moore, J., Chen, K., Lassaletta, A. D., Yi, C.-S., Tyson, J. J. and Sible, J. C. (2003). Hysteresis drives cell-cycle transitions in Xenopus laevis egg extracts. *Proc. Natl. Acad. Sci. USA* 100, 975-980. 10.1073/pnas.0235349100 12509509 PMC298711

[DEV204217C219] She, W., Grimanelli, D., Rutowicz, K., Whitehead, M. W. J., Puzio, M., Kotlinski, M., Jerzmanowski, A. and Baroux, C. (2013). Chromatin reprogramming during the somatic-to-reproductive cell fate transition in plants. *Development* 140, 4008-4019. 10.1242/dev.095034 24004947

[DEV204217C220] Shimotohno, A., Matsubayashi, S., Yamaguchi, M., Uchimiya, H. and Umeda, M. (2003). Differential phosphorylation activities of CDK-activating kinases in Arabidopsis thaliana. *FEBS Lett.* 534, 69-74. 10.1016/S0014-5793(02)03780-8 12527363

[DEV204217C221] Shimotohno, A., Umeda-Hara, C., Bisova, K., Uchimiya, H. and Umeda, M. (2004). The plant-specific kinase CDKF;1 is involved in activating phosphorylation of cyclin-dependent kinase-activating kinases in Arabidopsis. *Plant Cell* 16, 2954-2966. 10.1105/tpc.104.025601 15486101 PMC527191

[DEV204217C222] Simanis, V. and Nurse, P. (1986). The cell cycle control gene cdc2+ of fission yeast encodes a protein kinase potentially regulated by phosphorylation. *Cell* 45, 261-268. 10.1016/0092-8674(86)90390-9 3516412

[DEV204217C223] Simonini, S., Bemer, M., Bencivenga, S., Gagliardini, V., Pires, N. D., Desvoyes, B., van der Graaff, E., Gutierrez, C. and Grossniklaus, U. (2021). The Polycomb group protein MEDEA controls cell proliferation and embryonic patterning in Arabidopsis. *Dev. Cell* 56, 1945-1960.e7. 10.1016/j.devcel.2021.06.004 34192526 PMC8279741

[DEV204217C224] Simonini, S., Bencivenga, S. and Grossniklaus, U. (2024). A paternal signal induces endosperm proliferation upon fertilization in Arabidopsis. *Science* 383, 646-653. 10.1126/science.adj4996 38330116

[DEV204217C225] Smyth, D. R., Bowman, J. L. and Meyerowitz, E. M. (1990). Early flower development in Arabidopsis. *Plant Cell* 2, 755-767. 10.1105/tpc.2.8.755 2152125 PMC159928

[DEV204217C226] Sofroni, K., Takatsuka, H., Yang, C., Dissmeyer, N., Komaki, S., Hamamura, Y., Böttger, L., Umeda, M. and Schnittger, A. (2020). CDKD-dependent activation of CDKA;1 controls microtubule dynamics and cytokinesis during meiosis. *J. Cell Biol.* 219, e201907016. 10.1083/jcb.201907016 32609301 PMC7401817

[DEV204217C227] Soni, R., Carmichael, J. P., Shah, Z. H. and Murray, J. A. (1995). A family of cyclin D homologs from plants differentially controlled by growth regulators and containing the conserved retinoblastoma protein interaction motif. *Plant Cell* 7, 85-103. 10.1105/tpc.7.1.85 7696881 PMC160767

[DEV204217C228] Sorrell, D. A., Marchbank, A., McMahon, K., Dickinson, J. R., Rogers, H. J. and Francis, D. (2002). A WEE1 homologue from Arabidopsis thaliana. *Planta* 215, 518-522. 10.1007/s00425-002-0815-4 12111237

[DEV204217C229] Springer, P. S., Holding, D. R., Groover, A., Yordan, C. and Martienssen, R. A. (2000). The essential Mcm7 protein PROLIFERA is localized to the nucleus of dividing cells during the G1 phase and is required maternally for early Arabidopsis development. *Development* 127, 1815-1822. 10.1242/dev.127.9.1815 10751170

[DEV204217C230] Stallaert, W., Kedziora, K. M., Chao, H. X. and Purvis, J. E. (2019). Bistable switches as integrators and actuators during cell cycle progression. *FEBS Lett.* 593, 2805-2816. 10.1002/1873-3468.13628 31566708 PMC7881439

[DEV204217C231] Steinhardt, R. A. and Epel, D. (1974). Activation of sea-urchin eggs by a calcium ionophore. *Proc. Natl. Acad. Sci. USA* 71, 1915-1919. 10.1073/pnas.71.5.1915 4525301 PMC388353

[DEV204217C232] Strasburger, E. (1876). *Über Zellbildung und Zelltheilung ("On Cell Formation and Cell Division")*. Jena: Hermann Dabis. https://wellcomecollection.org/works/bankpd46

[DEV204217C233] Stricker, S. A. (1999). Comparative biology of calcium signaling during fertilization and egg activation in animals. *Dev. Biol.* 211, 157-176. 10.1006/dbio.1999.9340 10395780

[DEV204217C234] Sukawa, Y. and Okamoto, T. (2018). Cell cycle in egg cell and its progression during zygotic development in rice. *Plant Reprod* 31, 107-116. 10.1007/s00497-017-0318-x 29270910

[DEV204217C235] Sun, Y., Dilkes, B. P., Zhang, C., Dante, R. A., Carneiro, N. P., Lowe, K. S., Jung, R., Gordon-Kamm, W. J. and Larkins, B. A. (1999). Characterization of maize (Zea mays L.) Wee1 and its activity in developing endosperm. *Proc. Natl. Acad. Sci. USA* 96, 4180-4185. 10.1073/pnas.96.7.4180 10097184 PMC22441

[DEV204217C236] Swift, H. (1950). The constancy of desoxyribose nucleic acid in plant nuclei. *Proc. Natl. Acad. Sci. USA* 36, 643-654. 10.1073/pnas.36.11.643 14808154 PMC1063260

[DEV204217C237] Takahashi, I., Kojima, S., Sakaguchi, N., Umeda-Hara, C. and Umeda, M. (2010). Two Arabidopsis cyclin A3s possess G1 cyclin-like features. *Plant Cell Rep.* 29, 307-315. 10.1007/s00299-010-0817-9 20130883

[DEV204217C238] Takatsuka, H., Ohno, R. and Umeda, M. (2009). The Arabidopsis cyclin-dependent kinase-activating kinase CDKF;1 is a major regulator of cell proliferation and cell expansion but is dispensable for CDKA activation. *Plant J. Cell Mol. Biol.* 59, 475-487. 10.1111/j.1365-313X.2009.03884.x 19368694

[DEV204217C239] Takatsuka, H., Umeda-Hara, C. and Umeda, M. (2015). Cyclin-dependent kinase-activating kinases CDKD;1 and CDKD;3 are essential for preserving mitotic activity in Arabidopsis thaliana. *Plant J. Cell Mol. Biol.* 82, 1004-1017. 10.1111/tpj.12872 25942995

[DEV204217C340] Tao, P. and Nodine, M. D. (2019). Transcriptional activation of Arabidopsis zygotes is required for initial cell divisions. *Sci. Rep.* 9, 17159. 10.1038/s41598-019-53704-2 31748673 PMC6868190

[DEV204217C240] Tian, H. Q., Yuan, T. and Russell, S. D. (2005). Relationship between double fertilization and the cell cycle in male and female gametes of tobacco. *Sex. Plant Reprod* 17, 243-252. 10.1007/s00497-004-0233-9

[DEV204217C241] Townsley, F. M. and Ruderman, J. V. (1998). Proteolytic ratchets that control progression through mitosis. *Trends Cell Biol.* 8, 238-244. 10.1016/s0962-8924(98)01268-9 9695848

[DEV204217C242] Twell, D. (2011). Male gametogenesis and germline specification in flowering plants. *Sex. Plant Reprod.* 24, 149-160. 10.1007/s00497-010-0157-5 21103996

[DEV204217C243] Umeda, M., Shimotohno, A. and Yamaguchi, M. (2005). Control of cell division and transcription by cyclin-dependent kinase-activating kinases in plants. *Plant Cell Physiol.* 46, 1437-1442. 10.1093/pcp/pci170 16024551

[DEV204217C244] Underwood, C. J., Vijverberg, K., Rigola, D., Okamoto, S., Oplaat, C., den Camp, R. H. M. O., Radoeva, T., Schauer, S. E., Fierens, J., Jansen, K. et al. (2022). A PARTHENOGENESIS allele from apomictic dandelion can induce egg cell division without fertilization in lettuce. *Nat. Genet.* 54, 84-93. 10.1038/s41588-021-00984-y 34992267

[DEV204217C245] Van Beneden, E. (1883). Recherches sur la maturation de l'oeuf, la fe′condation et la division cellulaire. *Arch. Biol.* 4, 265-640.

[DEV204217C246] Van Leene, J., Hollunder, J., Eeckhout, D., Persiau, G., Van De Slijke, E., Stals, H., Van Isterdael, G., Verkest, A., Neirynck, S., Buffel, Y. et al. (2010). Targeted interactomics reveals a complex core cell cycle machinery in Arabidopsis thaliana. *Mol. Syst. Biol.* 6, 397. 10.1038/msb.2010.53 20706207 PMC2950081

[DEV204217C247] Vandel, L., Nicolas, E., Vaute, O., Ferreira, R., Ait-Si-Ali, S. and Trouche, D. (2001). Transcriptional repression by the retinoblastoma protein through the recruitment of a histone methyltransferase. *Mol. Cell. Biol.* 21, 6484-6494. 10.1128/MCB.21.19.6484-6494.2001 11533237 PMC99795

[DEV204217C248] Vandepoele, K., Raes, J., De Veylder, L., Rouzé, P., Rombauts, S. and Inzé, D. (2002). Genome-wide analysis of core cell cycle genes in Arabidopsis. *Plant Cell* 14, 903-916. 10.1105/tpc.010445 11971144 PMC150691

[DEV204217C249] Venta, R., Valk, E., Kõivomägi, M. and Loog, M. (2012). Double-negative feedback between S-phase cyclin-CDK and CKI generates abruptness in the G1/S switch. *Front. Physiol.* 3, 459. 10.3389/fphys.2012.00459 23230424 PMC3515773

[DEV204217C250] Verkest, A., Weinl, C., Inzé, D., De Veylder, L. and Schnittger, A. (2005). Switching the cell cycle. Kip-related proteins in plant cell cycle control. *Plant Physiol.* 139, 1099-1106. 10.1104/pp.105.069906 16286449 PMC1283750

[DEV204217C251] Voichek, Y., Hurieva, B., Michaud, C., Schmücker, A., Vergara, Z., Desvoyes, B., Gutierrez, C., Nizhynska, V., Jaegle, B., Borg, M. et al. (2023). Cell cycle status of male and female gametes during Arabidopsis reproduction. *Plant Physiol.* 194, 412-421. 10.1093/plphys/kiad512 37757882 PMC10756760

[DEV204217C252] Waldeyer-Hartz, H. (1888). Über Karyokinese und ihre Beziehungen zu den Befruchtungsvorgängen. *Arch. Mikroskopische Anat. Entwicklungsmechanik* 32, 1-122.

[DEV204217C253] Wang, G., Kong, H., Sun, Y., Zhang, X., Zhang, W., Altman, N., DePamphilis, C. W. and Ma, H. (2004). Genome-wide analysis of the cyclin family in Arabidopsis and comparative phylogenetic analysis of plant cyclin-like proteins. *Plant Physiol.* 135, 1084-1099. 10.1104/pp.104.040436 15208425 PMC514142

[DEV204217C254] Wang, H., Fowke, L. C. and Crosby, W. L. (1997). A plant cyclin-dependent kinase inhibitor gene. *Nature* 386, 451-452. 10.1038/386451a0 9087400

[DEV204217C255] Wang, Y., Cheng, Z., Huang, J., Shi, Q., Hong, Y., Copenhaver, G. P., Gong, Z. and Ma, H. (2012a). The DNA replication factor RFC1 is required for interference-sensitive meiotic crossovers in Arabidopsis thaliana. *PLoS Genet.* 8, e1003039. 10.1371/journal.pgen.1003039 23144629 PMC3493451

[DEV204217C256] Wang, Y., Hou, Y., Gu, H., Kang, D., Chen, Z., Liu, J. and Qu, L.-J. (2012b). The Arabidopsis APC4 subunit of the anaphase-promoting complex/cyclosome (APC/C) is critical for both female gametogenesis and embryogenesis. *Plant J. Cell Mol. Biol.* 69, 227-240. 10.1111/j.1365-313X.2011.04785.x 21910774

[DEV204217C257] Wang, Y., Hou, Y., Gu, H., Kang, D., Chen, Z.-L., Liu, J. and Qu, L.-J. (2013). The Arabidopsis anaphase-promoting complex/cyclosome subunit 1 is critical for both female gametogenesis and embryogenesis(F). *J. Integr. Plant Biol.* 55, 64-74. 10.1111/jipb.12018 23206231

[DEV204217C258] Williams, J. H. and Friedman, W. E. (2002). Identification of diploid endosperm in an early angiosperm lineage. *Nature* 415, 522-526. 10.1038/415522a 11823859

[DEV204217C259] Williams, J. H. and Friedman, W. E. (2004). The four-celled female gametophyte of Illicium (Illiciaceae; Austrobaileyales): implications for understanding the origin and early evolution of monocots, eumagnoliids, and eudicots. *Am. J. Bot.* 91, 332-351. 10.3732/ajb.91.3.332 21653390

[DEV204217C260] Woodard, J. W. (1956). DNA in gametogenesis and embryogeny in Tradescantia. *J. Biophys. Biochem. Cytol.* 2, 765-776. 10.1083/jcb.2.6.765 13398443 PMC2224002

[DEV204217C261] Wu, X., Xie, L., Sun, X., Wang, N., Finnegan, E. J., Helliwell, C., Yao, J., Zhang, H., Wu, X., Hands, P. et al. (2023). Mutation in Polycomb repressive complex 2 gene OsFIE2 promotes asexual embryo formation in rice. *Nat. Plants* 9, 1848-1861. 10.1038/s41477-023-01536-4 37814022 PMC10654051

[DEV204217C262] Xie, Q., Sanz-Burgos, A. P., Hannon, G. J. and Gutiérrez, C. (1996). Plant cells contain a novel member of the retinoblastoma family of growth regulatory proteins. *EMBO J.* 15, 4900-4908. 10.1002/j.1460-2075.1996.tb00870.x 8890163 PMC452227

[DEV204217C263] Xie, Q., Suárez-López, P. and Gutiérrez, C. (1995). Identification and analysis of a retinoblastoma binding motif in the replication protein of a plant DNA virus: requirement for efficient viral DNA replication. *EMBO J.* 14, 4073-4082. 10.1002/j.1460-2075.1995.tb00079.x 7664747 PMC394486

[DEV204217C264] Yadegari, R. and Drews, G. N. (2004). Female gametophyte development. *Plant Cell* 16 Suppl. 1, S133-S141. 10.1105/tpc.018192 15075395 PMC2643389

[DEV204217C265] Yang, C., Hamamura, Y., Sofroni, K., Böwer, F., Stolze, S. C., Nakagami, H. and Schnittger, A. (2019). SWITCH 1/DYAD is a WINGS APART-LIKE antagonist that maintains sister chromatid cohesion in meiosis. *Nat. Commun.* 10, 1755. 10.1038/s41467-019-09759-w 30988453 PMC6465247

[DEV204217C266] Yang, C., Sofroni, K., Wijnker, E., Hamamura, Y., Carstens, L., Harashima, H., Stolze, S. C., Vezon, D., Chelysheva, L., Orban-Nemeth, Z. et al. (2020). The Arabidopsis Cdk1/Cdk2 homolog CDKA;1 controls chromosome axis assembly during plant meiosis. *EMBO J.* 39, e101625. 10.15252/embj.2019101625 31556459 PMC6996576

[DEV204217C267] Yang, W., Wightman, R. and Meyerowitz, E. M. (2017). Cell cycle control by nuclear sequestration of CDC20 and CDH1 mRNA in plant stem cells. *Mol. Cell* 68, 1108-1119.e3. 10.1016/j.molcel.2017.11.008 29225038 PMC6013263

[DEV204217C268] Zamanian, M. and La Thangue, N. B. (1992). Adenovirus E1a prevents the retinoblastoma gene product from repressing the activity of a cellular transcription factor. *EMBO J.* 11, 2603-2610. 10.1002/j.1460-2075.1992.tb05325.x 1385776 PMC556735

[DEV204217C269] Zatulovskiy, E., Zhang, S., Berenson, D. F., Topacio, B. R. and Skotheim, J. M. (2020). Cell growth dilutes the cell cycle inhibitor Rb to trigger cell division. *Science* 369, 466-471. 10.1126/science.aaz6213 32703881 PMC7489475

[DEV204217C270] Zhang, J., Pai, Q., Yue, L., Wu, X., Liu, H. and Wang, W. (2022). Cytokinin regulates female gametophyte development by cell cycle modulation in Arabidopsis thaliana. *Plant Sci.* 324, 111419. 10.1016/j.plantsci.2022.111419 35995110

[DEV204217C271] Zhao, X., Bramsiepe, J., Van Durme, M., Komaki, S., Prusicki, M. A., Maruyama, D., Forner, J., Medzihradszky, A., Wijnker, E., Harashima, H. et al. (2017). RETINOBLASTOMA RELATED1 mediates germline entry in Arabidopsis. *Science* 356, eaaf6532. 10.1126/science.aaf6532 28450583

[DEV204217C272] Zhao, X., Harashima, H., Dissmeyer, N., Pusch, S., Weimer, A. K., Bramsiepe, J., Bouyer, D., Rademacher, S., Nowack, M. K., Novak, B. et al. (2012). A general G1/S-phase cell-cycle control module in the flowering plant Arabidopsis thaliana. *PLoS Genet.* 8, e1002847. 10.1371/journal.pgen.1002847 22879821 PMC3410867

